# Overexpression of POLA2 in hepatocellular carcinoma is involved in immune infiltration and predicts a poor prognosis

**DOI:** 10.1186/s12935-023-02949-z

**Published:** 2023-07-14

**Authors:** Long Liu, Qi Wang, Linjun Wu, Lele Zhang, Yuxi Huang, Haihua Yang, Le guo, Zheping Fang, Xuequan Wang

**Affiliations:** 1 Department of Hepatobiliary Surgery, Taizhou Hospital of Zhejiang Province, Zhejiang University, Linhai, 317000 Zhejiang China; 2 Department of Radiation Oncology, Taizhou Hospital of Zhejiang Province, Linhai, 317000 Zhejiang China; 3 Department of Medical Laboratory, School of Clinical Medicine, Ningxia Medical University, Yinchuan, 750004 China; 4 Taizhou Hospital Library, Wenzhou Medical University, Linhai, 317000 Zhejiang China; 5 Taizhou Hospital of Zhejiang Province, Wenzhou Medical University, Linhai, 317000 Zhejiang China; 6 Key Laboratory of Minimally Invasive Techniques & Rapid Rehabilitation of Digestive, System Tumor of Zhejiang Province, Zhejiang, China

**Keywords:** POLA2, Hepatocellular carcinoma, Prognostic, Immune infiltration, DNA polymerase α

## Abstract

**Background:**

Hepatocellular carcinoma (HCC) is the second malignancy worldwide. POLA2 initiates DNA replication, regulates cell cycle and gene repair that promote tumorigenesis and disease progression. However, the prognostic and biological function roles of POLA2 in HCC had not been conclusively determined.

**Methods:**

The expression levels and prognosis role of POLA1 and POLA2 in HCC were analyzed based on TCGA-LIHC database and recruited 24 HCC patients. Gene mutations were analyzed using “maftools” package. POLA2 and immune cells correlations were analyzed by TIMER. POLA2 co-expressed genes functional enrichment were evaluated using Metascape. The mRNA and protein level of POLA2 was detected in HCC cells and tissues. Cell migration, invasion, proliferation, cell cycle and HCC cell lines derived xenograft model were performed to investigate POLA2 biological function.

**Results:**

POLA2 was significantly high expressed in HCC than in normal liver tissue in both TCGA-LIHC and our collected HCC samples. In validation cohort, POLA2 significantly related to tumor differentiation, tumor size and Ki-67 (p < 0.05). In TCGA-LIHC cohort, overexpression of POLA2 predicted a low OS and associated with different clinical stages. Multivariate Cox regression showed overexpression of POLA2 effectively distinguished the prognosis at different T, N, M, stages and grades of HCC. POLA2 expression correlated with mutation burden, immune cells infiltration and immune-associated genes expression of HCC. Functional enrichment revealed that POLA2 co-expressed genes were linked to cellular activity, plasma membrane protein complex and leukocyte activity, immune response-regulated cell surface receptor signaling pathway, and immune response-regulated signaling pathway. Moreover, POLA2 was also positively co-expressed with some immune checkpoints (CD274, CTL-4, HAVCR2, PDCD1, PDCD1LG2, TIGIT, and LAG3) (p < 0.001). Gene knockdown revealed that POLA2 promoted proliferation, migration, invasion, and cell cycle of SMMC-7721 and HepG2. The HCC xenograft tumor model also demonstrated remarkably tumor size inhibition, tumor proliferation inhibtion and tumor necrosis promotion when POLA2 knockdown.

**Conclusions:**

POLA2 influenced immune microenvironment and tumor progression of HCC indicated that it might be a potential molecular marker for prognostic evaluation or a therapeutic target for HCC.

**Supplementary Information:**

The online version contains supplementary material available at 10.1186/s12935-023-02949-z.

## Background

Hepatocellular carcinoma (HCC) is the second most lethal malignancy worldwide, accounting for the majority (about 90%) of all primary liver cancers [1]. Many effective treatment modalities, including surgical resection of tumors, interventional techniques, and liver transplantation, are currently used for liver cancer [2]. However, they remain inadequate for end-stage or distant metastases liver cancers. Therefore, new strategies to improve the prognosis of patients with liver cancer are urgently needed.

DNA polymerases are enzymes that play a vital role in genome replication and repair. These proteins not only play a role in maintaining normal cell survival but also in the development and progression of cancers [3]. Therefore, depressing DNA polymerase expression should efficiently inhibit the proliferation of cancer cells. DNA polymerase family genes had been reported to function in developing different cancers [4, 5]. In HCC, DNA polymerases A, D, and E inhibition could arrest the growth of AFP-positive HCC cells [6]. POLQ knockdown interfered HCC development and progression by regulating cell proliferation, migration and apoptosis [7]. Elevated POLD1 expression promoted tumor progression and immunosuppressive microenvironment in HCC [8]. POLG polymorphisms were associated with the prognosis and mtDNA content in HCC patients [9]. A point mutation (T889C) in POLB increased progesterone receptor (PR) expression and intraperitoneal metastasis in gastric cancer [10]. Among these DNA polymerases, the alpha family (POLAs) consisting of POLA1 and POLA2, which initiate DNA synthesis and provide the substrate for more processive DNA polymerases, played a key role in cell proliferation [11, 12]. Researchers also found that POLA family genes promoted the progression of multiple cancers. POLA1 was a direct anticancer target for antitumor toxin CD437 [5]. POLA2 was involved in DNA double-strand breaks repair and the maintenance of gene stability [13], which were critical initiating factors in tumorigenesis. The prognosis of ovarian, gastric cancers, and glioblastoma multiforme tumors had been reported to be affected by POLA2 [13–15]. POLA2 also could act as a target antigen for tumor-infiltrating lymphocytes in persistent tumor regression [16]. These results prompted us to explore the effects of POLA2 on gene mutations and the biological functions of HCC. Moreover, in current years, the role of the tumor microenvironment has been gradually highlighted in tumor progress and treatment [17]. For example, among tumor-associated immune cells, the tumor-associated macrophage (TAM) has been shown to exacerbate cancer occurrence and malignant [18, 19]. However, the effect of POLA2 on infiltrating immune cells in the microenvironment of HCC remains to be investigated.

In this study, we investigated the role of POLAs in the prognosis of HCC and the effect of POLA2 overexpression on gene mutations in HCC. Moreover, we explored the correlation of POLA2 expression with immune cell infiltration and immune checkpoints in the HCC microenvironment. Finally, the effects of POLA2 on the proliferation, metastasis, invasion, and cell cycle were explored in vitro or in vivo experiments.

## Materials and methods

### Data and software

Fragments per kilobase of exon per million reads mapped (FPKM ) format RNA-seq read data and clinical data for HCC patients of the cancer genome atlas (TCGA) (https://portal.Gdc.cancer.gov/) LIHC datasets were obtained from the University of California, Santa Cruz Xena (https://xenabrowser.net/datapages/). R (version 4.03) and R studio (version 1.2.5042) software were used to perform statistical analysis.

### Analyze POLA1 and POLA2 expression in HCC

POLA2 expression levels in 424 HCC samples and 50 adjacent noncancerous tissue samples were analyzed by Wilcoxon testing of paired and unpaired groups, based on RNA-Seq data.

The mRNA level of POLA1 and POLA2 among different clinical staging system (T/N/M, grade, stage) were also analyzed by Kruskal–Wallis test and visualized by “RainCloudPlots” package (21). Gene expression date from HCCDB (Integrative Molecular Database of Hepatocellular Carcinoma, accessible at http://lifeome.net/database/hccdb) was used as additional data to analyzed PLOA1 and POLA2 expression in tumor and normal samples of HCC. ROC analysis by “pROC” package was used to evaluate the diagnose of HCC and normal liver tissues by POLA2 and POLA1 expression.

### Survival analysis of POLA1 and POLA2 in HCC

For survival analysis, the optimal cut-off values were used to split TCGA-LIHC into high and low groups for POLA1 and POLA2 based on expression values, survival time and survival status by the “survminer” package. The survival differences between low and high expression groups were then analyzed by the Kaplan-Meier and the log-rank tests.

Multivariate Cox regression analysis for OS prognosis was performed to analyze the significant correlation of POLA2 expression values combined with clinicopathological information including T, N, M, stage, grade of HCC patients. In addition, the role of high and low levels of POLA2 on OS in different clinicopathological stages and patients ages levels ( less than 45 years old, 45 : 69 years old, above 69 years old) was evaluated using Kaplan–Meier survival analysis [20]. R packages “ggplot2”, “survminer,“ and “survival” were used for the survival analysis and data visualization.

### HCC validation sample collection

Frozen HCC specimens and adjacent paracancer tissues from 24 HCC patients were collected from May 2022 to July 2022 at Taizhou Hospital, Zhejiang Province. The experimental protocol was approved by the Ethics Committee of Taizhou Hospital, Zhejiang Province. All frozen samples were pathologically diagnosed as HCC. A total of 12 male and 12 female patients were included. The ages of 24 patients ranged from 37 to 83 years. These HCC patients did not receive radiotherapy or chemotherapy before surgery. Exclusion criteria were vital organ dysfunction, bile duct cancer, and other organ tumors. Samples collected for the study were indicated in the signed informed consent form for subsequent clinical studies. All data were completely anonymous until we obtained them.

### Mutation data analysis of POLA2 in HCC

The somatic mutation data of 354 HCC patients were collected from TCGA database. To identify variations of mutated genes in POLA2 high and low expression groups, R package “TCGAmutations” and “maftools” R package were used for the mutation analysis [21, 22]. Furthermore, the “mafCompare” function in the “maftools” package was used to compare the mutation levels between high and low POLA2 expression groups. Additionally, the tumor mutation burden (TMB) in terms of per megabases for the TCGA-LIHC sample was calculated by the “tmb” function in the “maftools” package. The gene mutation influence on OS of these significant difference genes selected from high and low POLA2 expression group were also analyzed by cbioportal database (http://www.cbioportal.org/).

### Cell lines and cell transfection

Human normal hepatocyte line LO2 (HL-7702, l-02) cells which had morphological characteristics of hepatocytes were selected as normal hepatocyte control. All HCC cells (HepG2, SMMC-7721, MHCC-97, and SK-Hep1) and Human normal hepatocyte line LO2 cells were purchased from National Collection of Authenticated Cell Cultures (Shanghai, China). SMMC-7721 cells were cultured in RPMI-1640 (Invitrogen, Carlsbad, CA), and other cells were grown in DMEM (Invitrogen, Carlsbad, CA). All media were added with 10% fetal bovine serum (Invitrogen) and Penicillin-Streptomycin Solution (100X, Beyotime, China). All cells were cultured in an incubator at 37 °C and 5% CO_2_. GV493 vector (Promega Corp., Madison, Wisconsin, USA) containing small interfering RNA of POLA2 (si-POLA2) and negative control (NC) were designed and synthesized by Genechem (Shanghai, China) and transfected according to the instructions of the Lipofectamine 3000 kit (Thermo Fisher scientific) into cells.

### Quantitative real-time polymerase chain reaction (qRT-PCR)

Total RNA from cells and tissues was extracted using Trizol (Ambion, Invitrogen, USA). Reverse transcription into cDNA was completed by PrimeScript RT kit (TaKaRa, Dalian, China). The fluorescent Dye SYBR Green Master Mix (Applied Biosystems, Foster City, CA, USA) was detected in the ABI PRISM 7500 Sequence Detection System (Applied Biosystems, Inc.). The data were calculated using the 2-ΔΔCt method, and GAPDH was used as an endogenous control. The sequence of primers were shown as follows:

POLA2: forward, 5’- GGGATGTCCAGAGGCACTAACT-3’.

POLA2: reverse, 5’- CTTACAGGTCAGAACTTCTCGAATG-3’.

GAPDH: forward, 5’-ATAGCACAGCCTGGATAGCAACGTAC-3’.

GAPDH: reverse, 5’-CACCTTCTACAATGAGCTGCGTGTG-3’.

### Western blot assay

HepG2, SMMC-7721 cells and collected tissues were assayed using RIPA (radio-immunoprecipitation assay) buffer. BCA was used to measure total protein concentration. Proteins were then separated by 10% SDS-PAGE and transferred to PVDF (provided by Bio-Rad, Hercules, USA) membranes. The membranes were blocked with 5% nonfat milk and incubated with mono-antibodies against POLA2 (Absin, abs138967, 70KDa), GAPDH (Proteintech, 1E6D9, 36 KDa) and PD-L1 (Huabio, ET1701-41, 33 KDa). Afterward, the membranes were washed with TBST and incubated with secondary antibodies HRP* Goat Anti Mouse IgG (H + L) ( Immunoway, RS0001, China) or HRP* Goat Anti-Rabbit IgG (H + L) (RS0002, Immunoway, China). The results were measured with an enhanced chemiluminescence system. The imageQuant LAS500 was provided by GE Health Care (Fairfield, America). Chemiluminescent HRP Substrate was obtained from Millipore Corporation (Billerica, America). The intensity of the protein bands was determined by ImageJ software. GAPDH protein was used for normalization.

### Detection of function roles of POLA2 on HCC cells

Cell viability was detected by CCK-8 method (Beyotime, Cat No.C1052) and measured using a Multiskan FC Microplate Photometer (Thermo Fisher Scientific, Waltham, MA) at 450 nm absorbance. In colony formation assay, about 700 logarithmic growth phase cells per well were inoculated in culture dishes and cultured for about 10–14 days. Change the culture medium every three days, then 4% paraformaldehyde and 0.5% crystal violet staining solution were used to fixed and stained the cloned cells. Wound healing assay was performed as follows. 1*10^5 cells/well were inoculated into 6-well plates and incubated for 12 h to allow cells to adhere to the wall. Then, POLA2-sh and negative control were added to the culture medium according to the transfection protocol. After 24 h interference, a “cross-shaped” wound was made by a 200 µL pipette tip. Then washed gently with PBS three times, the cells were incubated in RIPM1640 and DMEM medium containing 2% FBS for another 24 h. The final images were taken under a phase-control microscope (Thermo, 100 X, microscope camera model). Transwell assay was utilized to examine the migration and invasion ability of cells. Chambers which were pre-coated with diluted matrigel (Corning, USA) (for invasion) or not (for migration) were inserted into 24-well plates. 5*10^4 treated cells in 200 µL serum-free medium were planted into the upper chamber, while 800 µL FBS-sufficient medium was added below the chamber. After 48-hour incubation (for migration) or longer (for invasion), cells on the bottom of chamber were immobilized and stained with Wright-Giemsa dye.The number of invading cells and migration cells was counted in three randomized regions. In cell cycle assay, the cells were stained using flow cytometry (BD FACSMelody, USA). The cells were stained with the Cell Cycle Assay Kit (Beyotime, China) 48 h after transfection, and flow cytometry (BD FACSMelody, USA) was used to assess the cell cycle. All experiments were replicated three times.

### Tumorigenesis studies

The BALB/c-nu mice (Shanghai SLAC Laboratory Animal Co., Ltd., China) used in this study were female nude mice, 6 weeks old, weighing between 18 and 22 g. They were housed in an SPF animal house at an ambient temperature of 26–28 °C and a humidity of 40–60%, and were housed in sterilized plastic cages with sealed air filtration devices. Water, food and bedding were sterilized and bedding was changed at least once a week. A total of 2*10^6 cells in 100 µl of PBS (suspended in 0.1 ml of PBS) were inoculated subcutaneously into the right abdomen of each mice. The tumor diameter of each mice was measured every 3 days. Tumor volume (mm³) was calculated as follows, V = (length * width^2)/2 was calculated. The mice were euthanized at 3 week and the tumors were surgically removed for IHC examination. This study was conducted in strict accordance with the guidelines of the Animal Experimentation Ethics Committee of Zhejiang Taizhou Hospital.

### Immunohistochemical staining (IHC)

Tumor tissue was paraffin-embedded and cut into 4-µm cross-sections. After dewaxing, antigen repair and blocking, sections were incubated with anti-POLA2 (Proteintech, Wuhan, China) and anti-ki-67 (Proteintech, Wuhan, China) for 2 h at 37 °C. The tissue was then incubated with biotin-labeled goat anti-rabbit secondary antibody for 20 min, followed by incubation with HRP-labeled streptavidin for 10 min at 37 °C. After washing with PBS, the nuclei were stained using hematoxylin solution.

### Analysis of infiltrating immune cells in HCC

Immune infiltration analysis of TCGA-LIHC patients was driven by the xCell algorithm and data from TIMER 2.0 database (http://timer.cistrome.org/) [23]. Pearson tests were performed to explore the correlation between POLA2 and the infiltrating of 39 immune cells types for HCC patient. The correlation between POLA2 expression levels and immune cells infiltrating levels were visualized by “corrplot” package. The “ggstatsplot” package was used to visualize POLA2 expression and the top correlated immune infiltration cells with Pearson statistical tests. POLA2 expression level in blood cell were further analyzed by Human Protein Atlas (HPA) website based on RNA-seq data from HPA dataset, Monaco dataset and Schmiedel dataset.

### Analysis of immune cells associated genes and checkpoints with POLA2 in HCC

The immune cell-specific genes were collected from a published study that contained 782 immune-related genes for 28 types of specifically labeled intratumoral immune cells [24]. The correlation of POLA2 and these immune-related genes or POLA1 were analyzed by Pearson correlation analysis. Genes that significantly co-expressed with PLOA2 in HCC were displayed (|R|>0.2, p < 0.001), and the top co-expressed genes were symbol displayed (|R|>0.2, p < 0.001). Metascape [20, 25] was used to analyze the potential function of POLA2 co-expressed immune related genes. The expression levels of eight representative immune checkpoint genes (CD274, HAVCR2, PDCD1, CTLA4, LAG3, SIGLEC15, TIGIT, and PDCD1L were selected to explore the co-expression relationship with POLA2.

## Results

### The differential expression of POLA1 and POLA2 in HCC and normal liver tissue

**Fig. 1:**
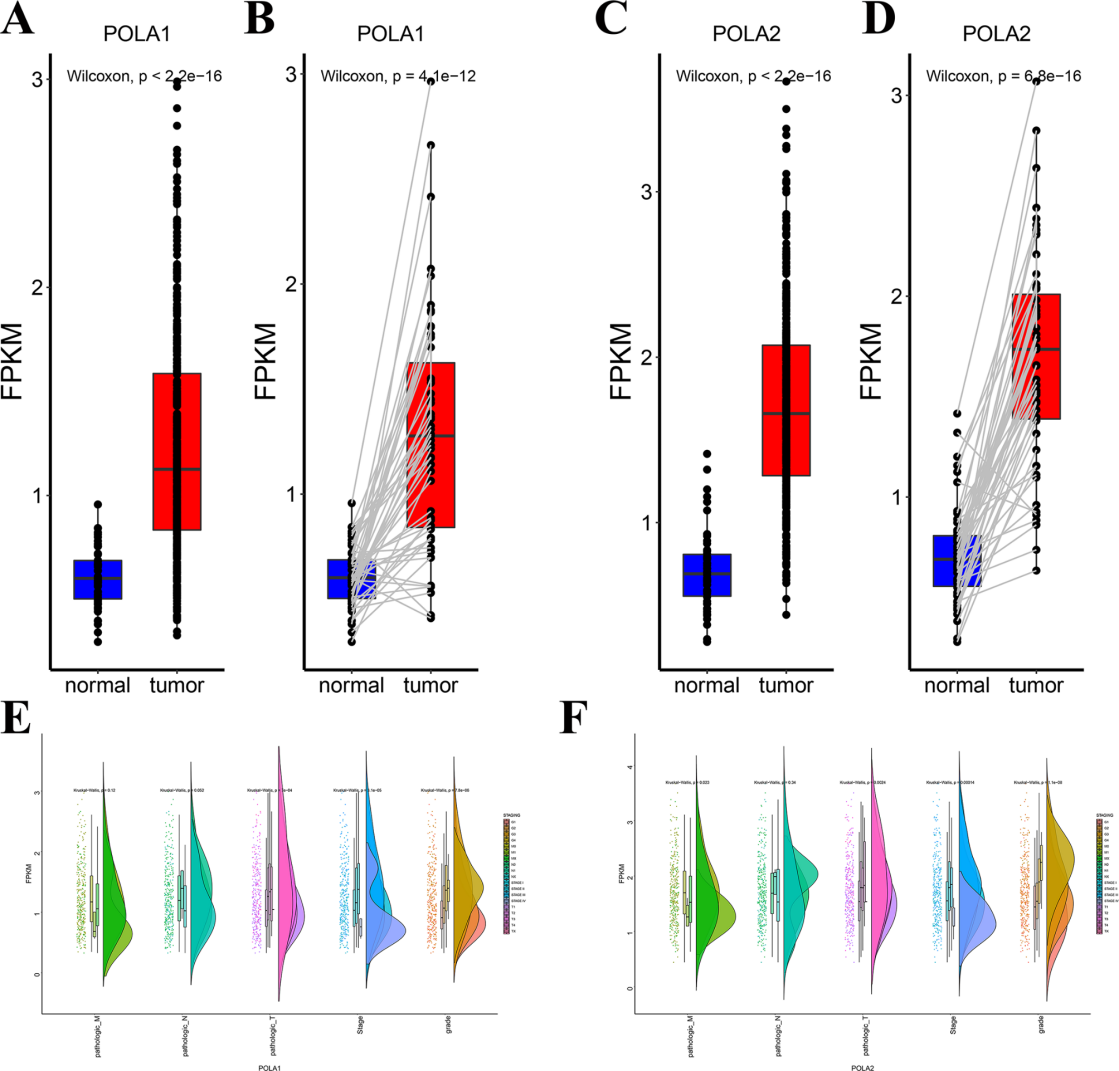
Expression analysis of POLA1 and POLA2 in HCC from TCGA database. The POLA1 expression in HCC and normal liver tissues (total samples in **A**; paired samples in **B**); The POLA2 expression in HCC and normal liver tissues (total samples in **C**, paired samples in **D**); Expression analyzed by Kruskal–Wallis test and “rainclouds” package for POLA1 (**E**) and POLA2 (**F**) in HCC patients with different clinicopathologic stages

To investigate the role of POLA1 and POLA2 in HCC, we first analyzed POLA1 and POLA2 mRNA or protein levels in 50 paired samples and 424 total samples from patients with HCC based on TCGA. The results revealed that the expression of POLA1 and POLA2 were significantly elevated in tumour tissues compared with in normal samples in total TCGA cohort (Fig. 1A/C). We further analyzed the paired samples (both tumor and normal tissues came from same patients) and found that over 95% of patients had higher expression of POLA1 and POLA2 in tumor tissue than in normal tissue (Fig. 1B/D). And data from HCCDB also demonstrated that both POLA1 and POLA2 was upregulated in HCC (Figure S1). We then examined the expression difference of POLA1 and POLA2 among different clinicopathologic features of HCC. The higher POLA1 and POLA2 expression values was observed to be associated with T-stage, clinical stage and grade. In addition, POLA2 was also associated with M-stage in patients with HCC (Fig. 1E-F).

### Survival prognostic analysis of POLA1 or POLA2 in HCC

**Fig. 2:**
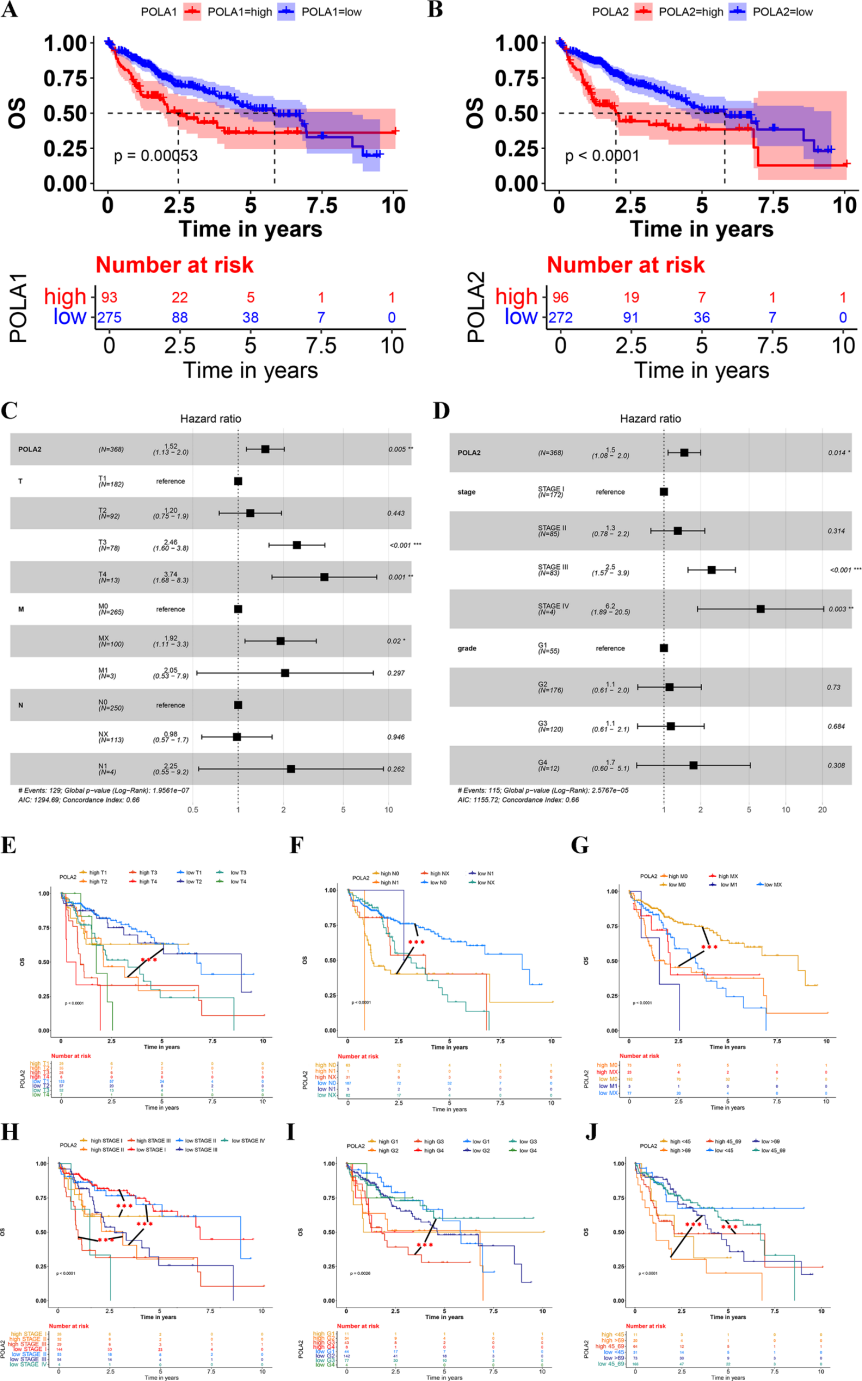
Comprehensive analysis of the impact of POLA1/2 expression and clinicopathological on HCC prognosis. Kaplan-Meier curves of the high and low expression groups of POLA1 (**A**) and POLA2 (**B**) in HCC. (**C**-**D**) Multivariate Cox analyses of POLA2 and (T, N, M, stage, and grade) correlations. (**E**-**J**) Survival analysis of POLA2 and T, N, M, stage, grade subgroups in HCC.

Cancer classification of HCC based on different stage system or other clinical information can stratify patient survival and provide more accurate treatment management and improve overall survival rate [26, 27]. To evaluate the survival prognostic impact, HCC cases were divided into high and low groups by their POLA1 and POLA2 mRNA levels. The results revealed that high levels of POLA1 and POLA2 in HCC significantly reduced the overall survival time (Fig. 2A-B). The median overall survival time were 2.46 years and 5.84 years for high and low expression groups of PLOA1. While for PLOA2, the median overall survival time were 1.98 years and 5.37 years for high and low expression groups respectively. By correlation analysis, we found that POLA1 and POLA2 has a significant coexpression correlation (Figure S2A), and this correlation influenced each other’s prediction of overall survival time in hepatocellular carcinoma (Figure S2C). Then ROC analysis showed that POLA2 (AUC 0.961) had better ability to distinguish between hepatocellular carcinoma and normal tissues than POLA1 (AUC 0.884) (Figure S2B).

Multivariate COX regression analysis showed that POLA2, T3/4 staging (p = 0.001), M staging (p = 0.02), Stage III/IV (p < 0.001; p = 0.003) could be used as independent prognosis factors of HCC (Fig. 2C-D). To explore more detailed impact of these factors on prognosis of HCC patients, we used Kaplan-Meier analysis to explore the prognosis of POLA2 expression group combine with T-stage, M-stage, N-stage, stage, pathological grade, or prognosis age of patients respectively (p < 0.001) (Fig. 2E-J). POLA2 showed significant prognosis role in all tested clinical groups. In addition, among all these different groups, there were significant OS differences between the POLA2 high and low HCC patients in the same clinical groups. As the results shown, for HCC T1 group, high POLA2 patients exhibited a poorer survival prognosis compared with low POLA2 patients (Fig. 2E). This significant prognosis also found in N0 stage (Fig. 2F), M0 stage(Fig. 2G), StageI, StageII, StageIII (Fig. 2H), Grade 3 (Fig. 2I) and patients above 65 years old (Fig. 2J). Due to the insufficient number of patients included, though in some clinical groups, POLA2 high and low expression patients did not show significant differences, but the survival curve also showed a trend of differences. In conclusion, high expression of POLA2 in HCC tissues would significantly reduced the overall survival time of HCC patients, and the expression level of POLA2 might be an effective marker for prognostic assessment of HCC patients. These results shown that POLA2 significantly affected the prognosis of patients with HCC and may provide higher efficacy in predicting the prognosis of HCC.

### Verification of POLA2 prognostic value in collected samples

**Fig. 3:**
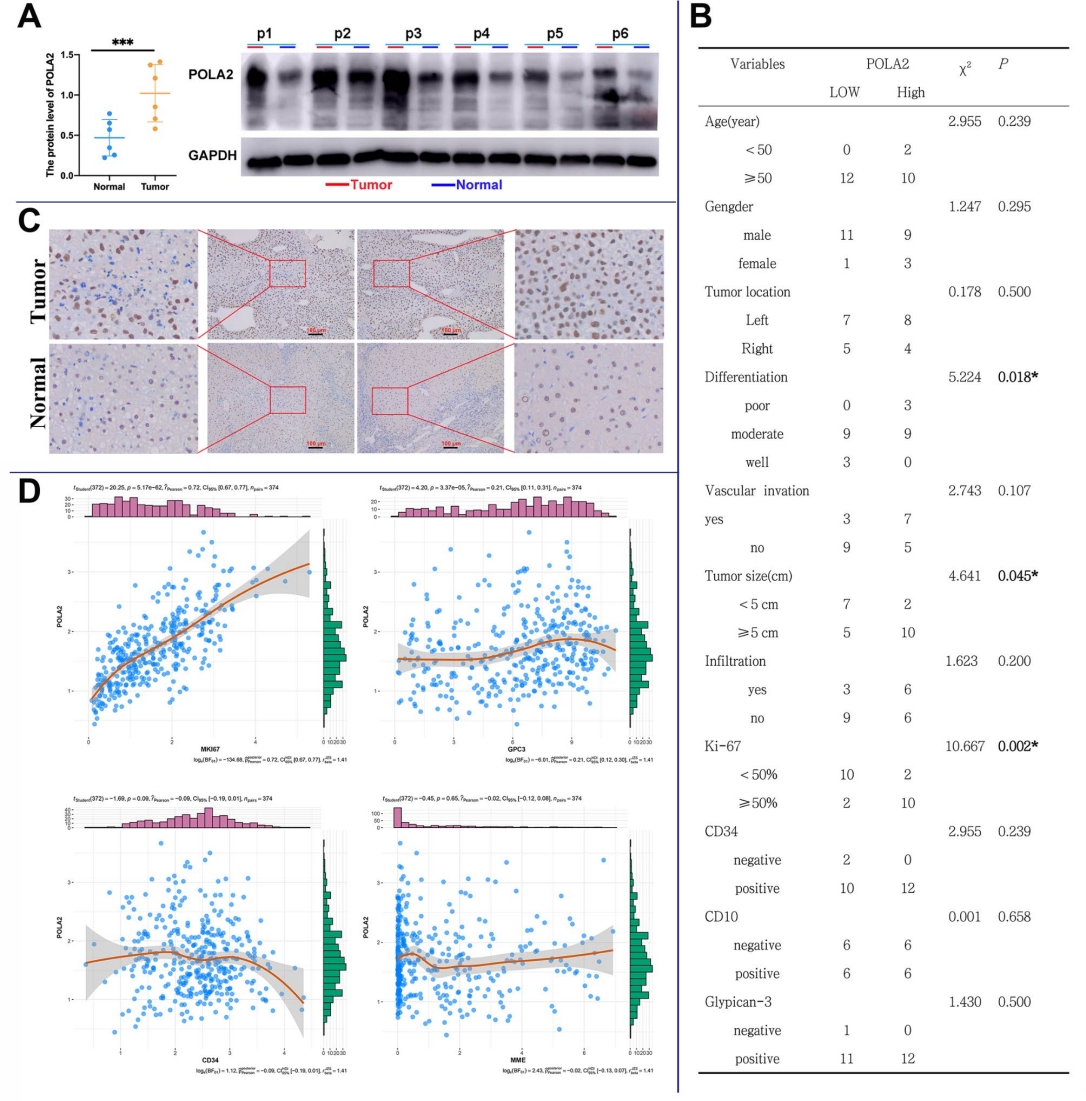
Correlation of POLA2 expression levels and clinicopathological factors in HCC samples. (**A**) Western blot detection of POLA2 expression in tissues; (**B**) Correlation analysis between POLA2 and clinicopathological factors; (**C**) Representative IHC images on the HCC tissue probed with the anti-POLA2 antibody (scale bars = 100 μm respectively) are shown. (**D**) Correlation analysis between POLA2 and MKI67, GPC3, CD34, MME based on TCGA database

The elevated level of POLA2 was also detected in 6 HCC samples compared with the adjacent non-tumor tissues (Fig. 3A). The correlation analysis of mRNA levels of POLA2 and clinicopathological factors in 24 HCC tissues showed that POLA2 was notably correlated with tumor differentiation level (p = 0.018), tumor size (p = 0.045) and Ki-67 (p = 0.002) in HCC (Fig. 3B). Besides, immunohistochemical (IHC) staining showed that POLA2 expression was significantly increased in HCC tissues compared to matched normal liver tissues (Fig. 3C). Based on TCGA database analysis, POLA2 was strongly correlated with MKI67 (Ki-67, p < 0.001) and GPC3 (p < 0.001) expression in HCC (Fig. 3D). Due to our clinical data’s small sample size, GPC3 does not show a significant relationship with POLA2 in our validation HCC cohort. These results not only verified the elevated level of POLA2 expression in HCC tissues, but also convincingly confirmed the correlation between POLA2 and clinicopathological factors of HCC.

### Correlation of POLA2 expression levels with gene mutations in HCC

**Fig. 4:**
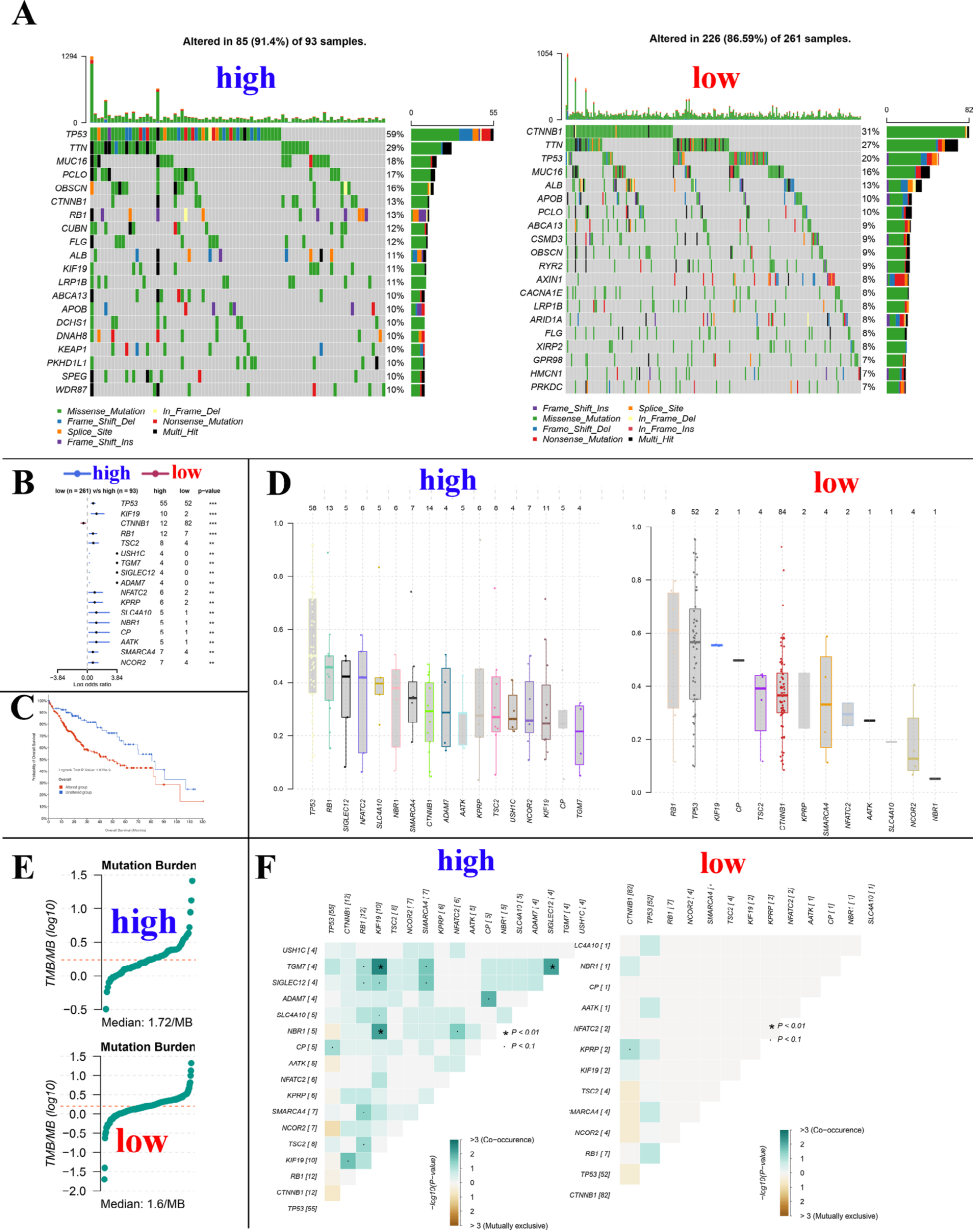
Effect of POLA2 on gene mutations in HCC. (**A**) Top 20 mutated genes in POLA2 high and low expression group. (**B**) Significant different mutation genes in POLA2 low and high expression groups. (**C**) Kaplan-Meier curves for HCC patiernts with or without mutations of above significant different mutation genes. (**D**) The variant allele frequency (VAF) of genes in POLA2 high expression group and POLA2 low expression group. (**E**) TMB value for HCC patients in POLA 2 high expression group and POLA2 low expression group. (**F**) Analysis mutation concurrence in the different mutated genes between POLA2 high and low expression groups

POLA2 plays a vital role in DNA repair and genomic stability. In addition, Gene mutations play an important role in cancer development. Therefore, we analyzed the mutation relationship with POLA2 expression. The results showed that the mutation rated of the top 20 mutated genes in the POLA2 high and low expression groups 91.4% and 86.59%, respectively (Fig. 4A). The top five genes in the high expression group were TP53, TTN, MUC16, PCLO, and OBSCN, while in the low expression group were CTNNB1, TTN, TP53, MUC16, and ALB (Fig. 4A). Between the high and low expression group of POLA2, total 17 genes showed significant different mutation rates (p < 0.01), and only CTNNB1 showed a high mutation rate in the low group (Fig. 4B). As we all know, mutation of these important oncogenes is the initiating factor of cell carcinogenesis, and it has been reported that mutation of TP53 and RB1 were closely related to the development and malignant progression of liver cancer, thus we speculate that POLA2 might promote the progression of liver cancer by promoting the mutation of oncogenes such as TP53 and RB1 [28]. In addition to this, the mutation rate of CTNNB1 was significantly higher in POLA2 low expression HCC, and we know that CTNNB1 encodes b-catenin, a key intracellular transducer of the Wnt signalling pathway that regulates liver physiology and zonation [29, 30]. Several studies have shown that HCCs with mutations in CTNNB1 display a specific phenotype of well-differentiated tumours with a micro-wart-like, pseudoglandular structural pattern [4, 31], which is consistent with HCC patients with low expression of POLA2 demonstrating a better prognosis. Survival analysis also showed that patients owned mutation of these 17 gene genes has significantly lower median survival time compare with wide type patients (p < 0.001). The median overall time is 51.25 (35.74 -NA) and 80.68 (69.51-NA) months for these altered and unaltered groups (Fig. 4C). The detailed variant allele frequency (VAF) of the difference mutated genes in high and low expression group were showed in Fig. 4D. The tumor mutational burden (TMB) also showed a positive correlation with POLA2 expression, and the TMB for higher and low expression groups was 1.72 MB and 1.6 MB, respectively (Fig. 4E). In POLA2 high expression group showed more significant mutation co-occurrence of different mutated genes (KIF19/TGM7, TGM7/SIGLEC12 and NBR1/KIG19, p < 0.05) (Fig. 4F). These results suggest that POLA2 were closely related gene mutations in HCC. This might also be one reason for the poor prognosis of HCC with high level of POLA2.

### The expression level regulation of POLA2 in HepG2 and SMMC-7721 cells

**Fig. 5:**
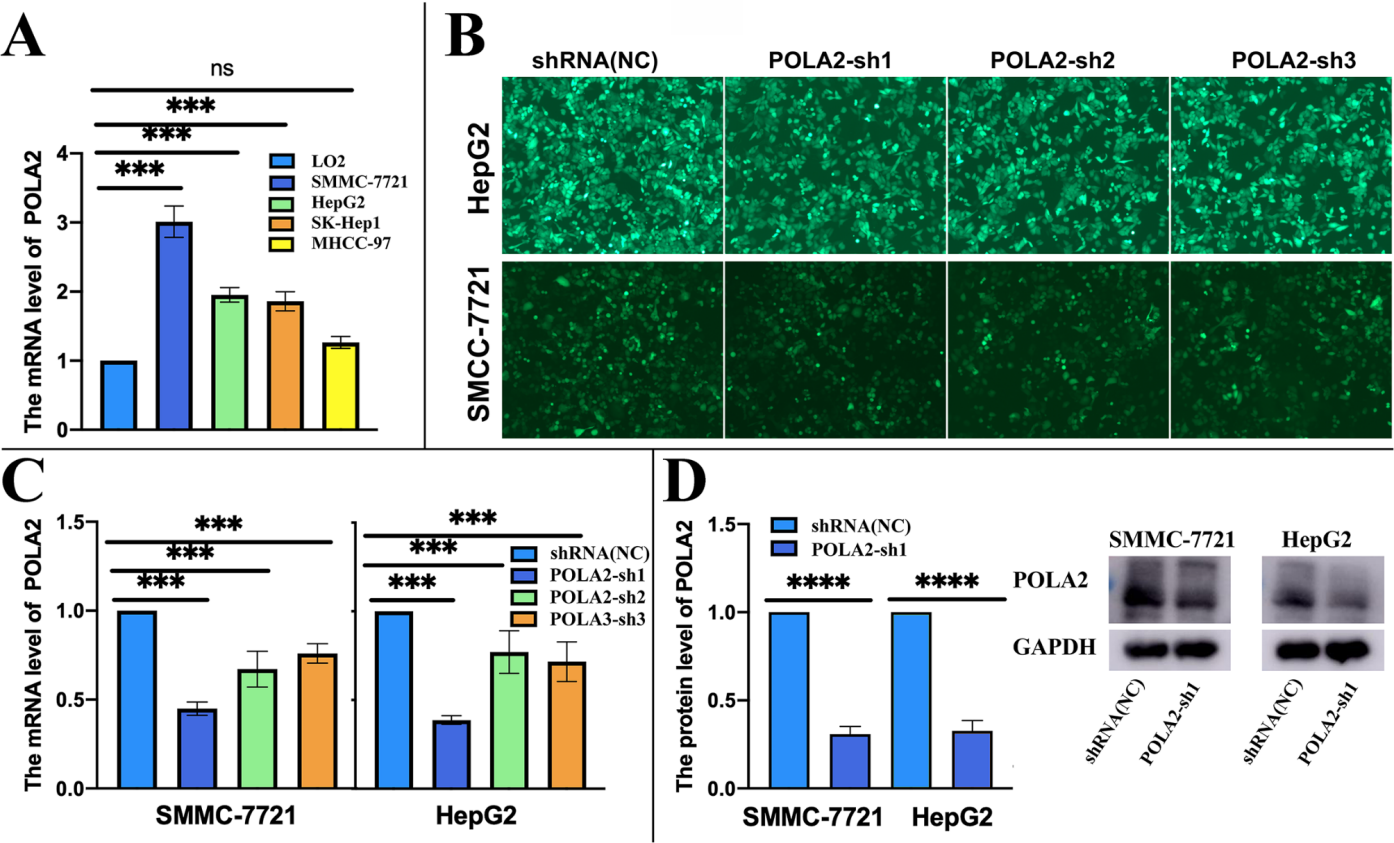
Expression levels of POLA2 in HCC cell lines. (**A**) The mRNA level of POLA2 in HepG2, SMMC-7721, MHCC-97, and SK-Hep1; (**B**) Fluorogram of GFP + POLA2-sh showed a high transfection efficiency in HepG2 and SMMC-7721 (DAPI for nuclear staining); POLA2 protein and mRNA were detected by qPCR (**C**) and Western blot assay (**D**) after HepG2 and SMMC-7721 cells transfected with POLA2sh.

Compared with the human-derived hepatocyte line LO2, the expression levels of POLA2 in SMMC-7721, HepG2, SK-Hep1, and MHCC-97 were all significantly overexpressed (Fig. 5A). We then down regulated the expression level of POLA2 in SMMC-7721 and HepG2 cells, since they were the top two POLA2 expression HCC cell lines we detected. GFP-tagged POLA2-sh and negative controls demonstrated the transfection efficiency of lentivirus, in HepG2 and SMMC-7721 cells. Meanwhile, we could see that transfection of POLA2-sh inhibited the proliferation of HepG2 and SMMC-7721 to different degrees. (Fig. 5B). After selection, POLA2-sh1 was chose for the follow-up experiments for its high knockdown efficiency (Fig. 5C). This transfection significantly reduced POLA2 protein level in HCC cells (Fig. 5D).

### Effect of POLA2 on the biological functions of HCC

**Fig. 6:**
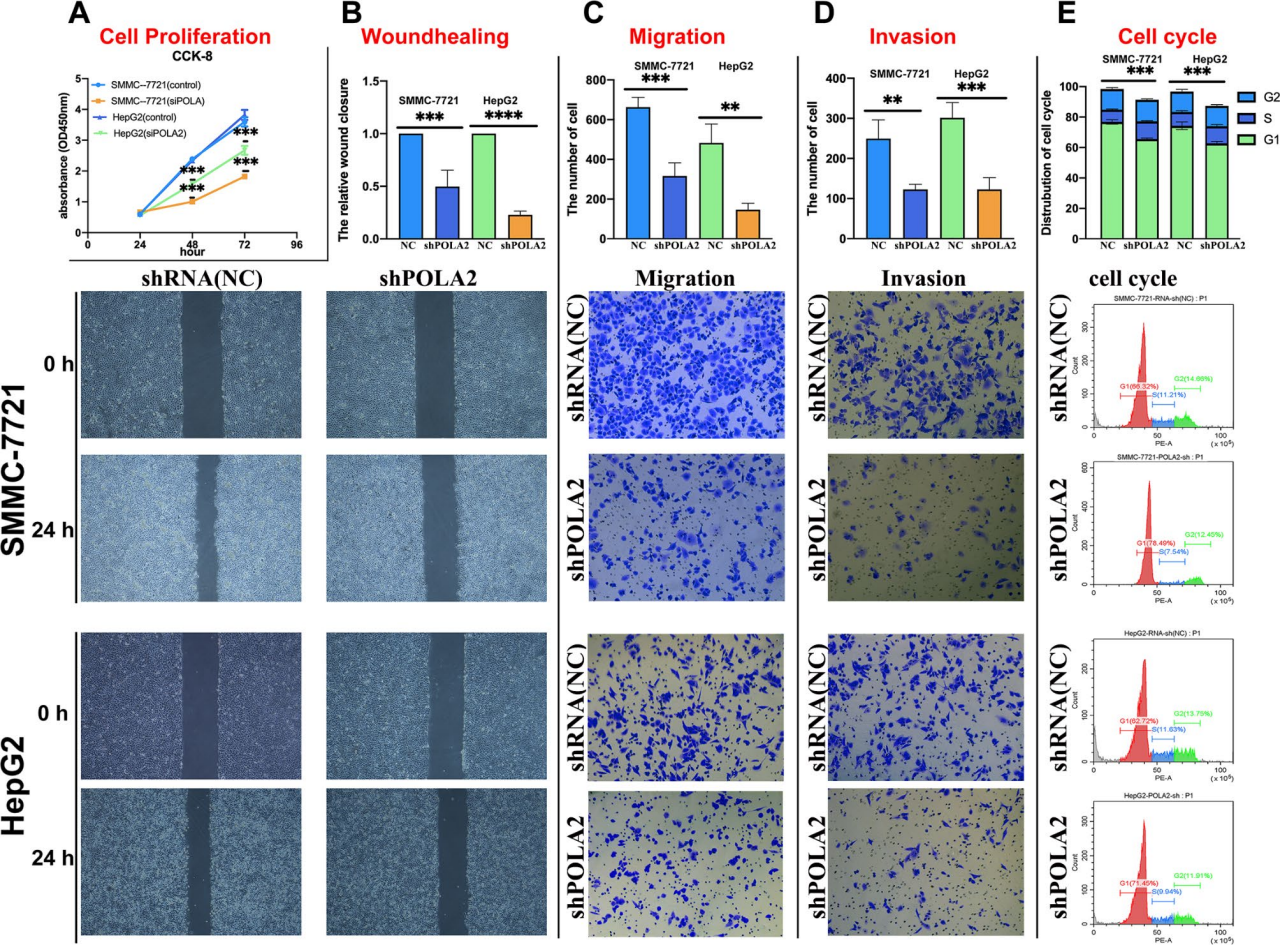
Effect of POLA2 on the biological functions of SMMC-7721 and HepG2. CCK-8 cell proliferation assay (**A**), scratch assay (**B**), Transwell cell migration (**C**), cell invasion assay (**D**), and flow cytometric assay of the cell cycle (**E**)

The viability of HepG2 and SMMC-7721 were reduced after transfected with POLA2sh, detected by CCK-8 assay. (Fig. 6A). The proliferation ability of HepG2 and SMMC-7721 was also significantly inhibited after POLA2 knock down through colony formation analysis (Figure S3). The scratch assay showed POLA2 knockdown significantly inhibited the wound healing ability of HepG2 and SMMC-7721 cells (Fig. 6B). Through Transwell cell migration and cell invasion assay, POLA2 knockdown showed significantly reduced HepG2 and SMMC-7721 migration and invasion ability (Fig. 6C-D). Finally,G1-phase ratios of the cell cycle were observably increased after reducing POLA2 expression levels in HepG2 and SMMC-7721, suggesting that POLA2-sh impeded the cell cycle process (Fig. 6E). Our experiments showed that aberrant expression of POLA2 accelerated the malignant process of tumor-related biological functions by affecting tumor proliferation, migration, invasion and cell cycle, which in turn mediated poor prognosis in patients with HCC.

**Fig. 7:**
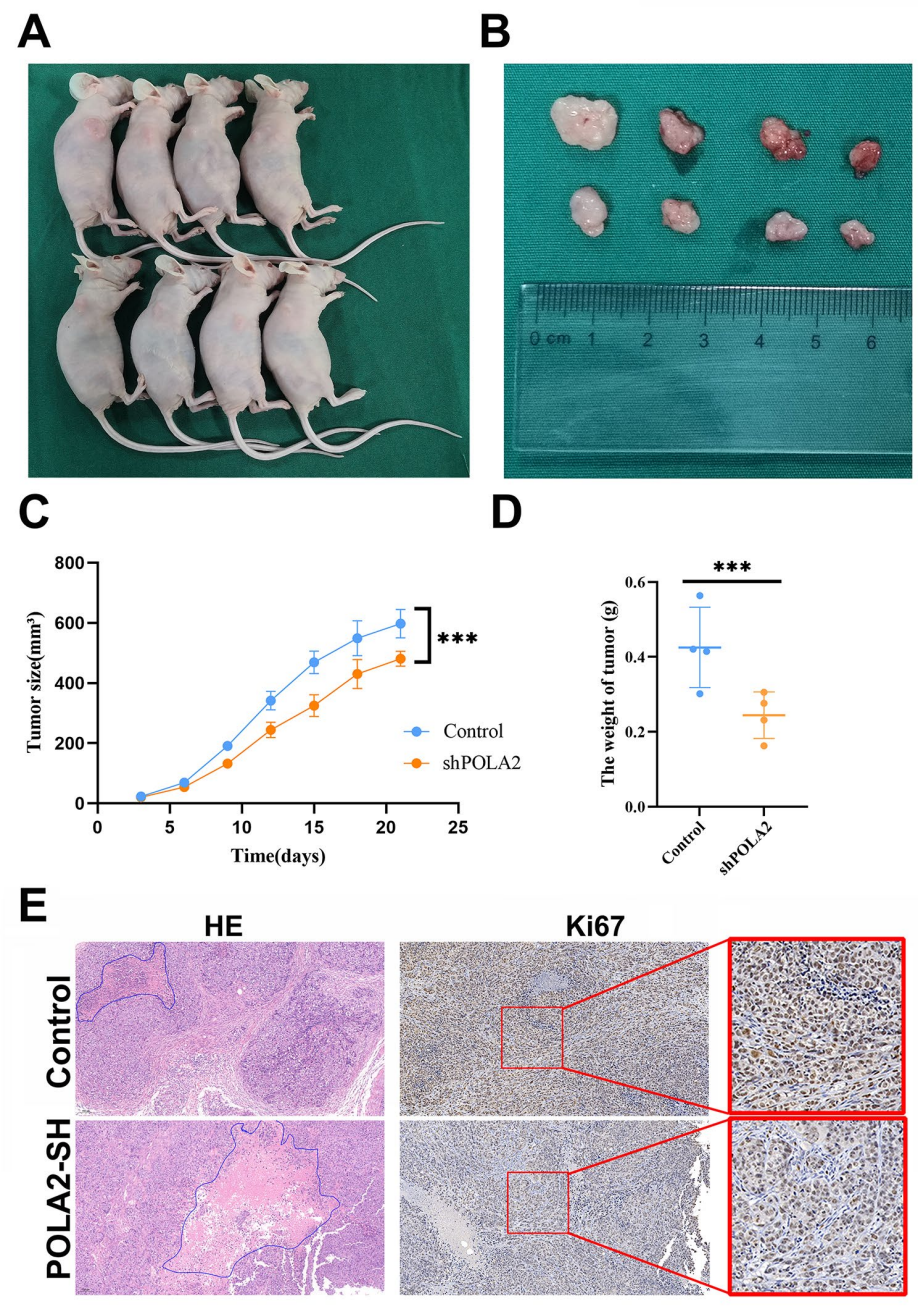
POLA2 expression influences tumor size, tumor proliferation and tumor necrosis in xenograft tumor model. (**A**) Tumor-bearing BALB/c-nu mice, (**B**) tumor volume, (**C**) tumor size and (**D**) tumor weight using HepG2 cells in a subcutaneous xenograft model. (**E**) Two representative tissue section images of control and POLA2 knockdown subcutaneous xenografts for Ki-67 measured using immunohistochemistry and tissue necrosis

To further understand the function of POLA2 in HCC, we established a HCC xenograft tumor model. HepG2 cells stably transfected with POLA2sh were transplanted into nude mice (Fig. 7A). Compared to NC group, POLA2-sh group significantly suppressed HepG2 tumor growth. Visual inspection (Fig. 7B-C) and weights of the dissected tumors (Fig. 7D) further demonstrated the remarkably tumor suppressive effects for inhibitation of POLA2. Immunohistochemical assays showed that the expression level of Ki-67 which was closely related to tumor proliferation, was significantly lower in the POLA2sh group compared to the negative control group (Fig. 7E). Consistently, HE staining of tumor tissues revealed a larger area of necrosis in POLA2sh than in the negative control group, which demonstrated that low levels of POLA2 suppress the survival of HCC cells. (Fig. 7F).

### POLA2 expression influences the immune cells infiltration of HCC

**Fig. 8:**
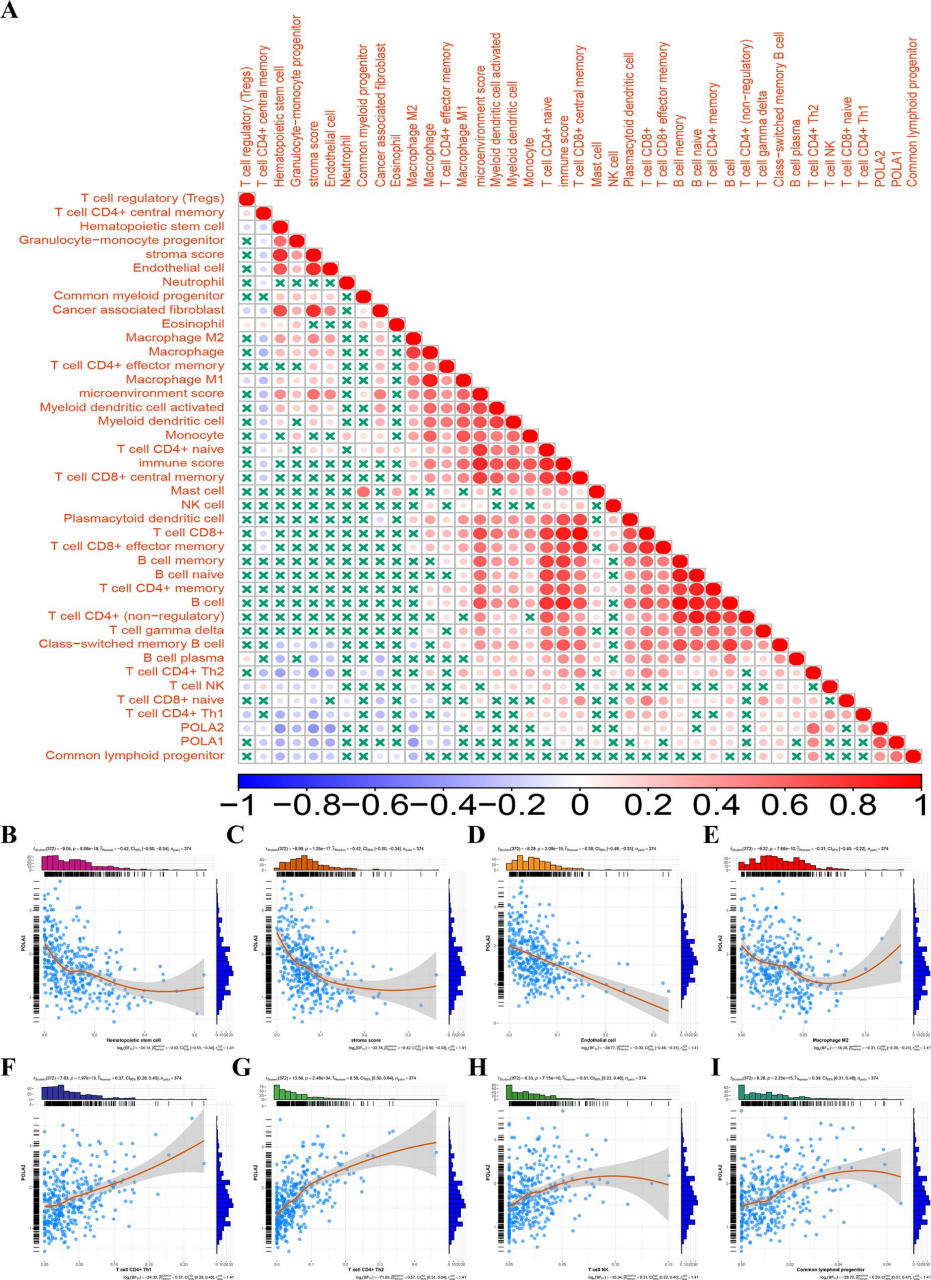
Association of POLA2 on infiltration of immune cells in HCC. (**A**) Relationship between POLA2 and infiltrating immune cells; (**B**-**I**) correlation analysis between POLA2 and different immune cells (T cell CD4 + Th1 (**B**), T cell CD4 + Th2 (**C**), T cell NK (**D**), common lymphoid progenitor (**E**), hematopoietic stem cells (**F**), stromal scores (**G**), endothelial cells (**H**) and macrophages M2 (**I**))

The infiltrating immune cells in the tumor microenvironment are linked to tumorigenesis, progression, and treatment in HCC [32]. Therefore, we further explored the correlation between POLA2 and infiltrated immune cells by TIMER (Fig. 8A). The expression levels of POLA2 and the infiltration levels of T cell CD4 + Th1, T cell CD4 + Th2, T cell NK and common lymphoid progenitor were significantly positively correlated (p < 0.01) (Fig. 8F-I). However, hematopoietic stem cells, stromal scores, endothelial cells, and macrophages M2 shown a significantly negative correlation with POLA2 expression (p < 0.01) (Fig. 8B-E). The results suggested that POLA2 might affect the prognosis of tumor patients also by influencing immune cells infiltration in HCC. Immune cell type expression data from HPA, Monaco, and Schmiedel dateset further indicated that IGF2BP2 was extensively expressed in most immune cells (Figure S4). These results may reflect the widespread expression of POLA2 in immune cells in liver cancer patients tumor tissue. Therefore intervening in POLA2 expression may affect immnue cell infiltration in liver cancer tissues.

### POLA2 affects immune cell related genes and immune checkpoint

**Fig. 9:**
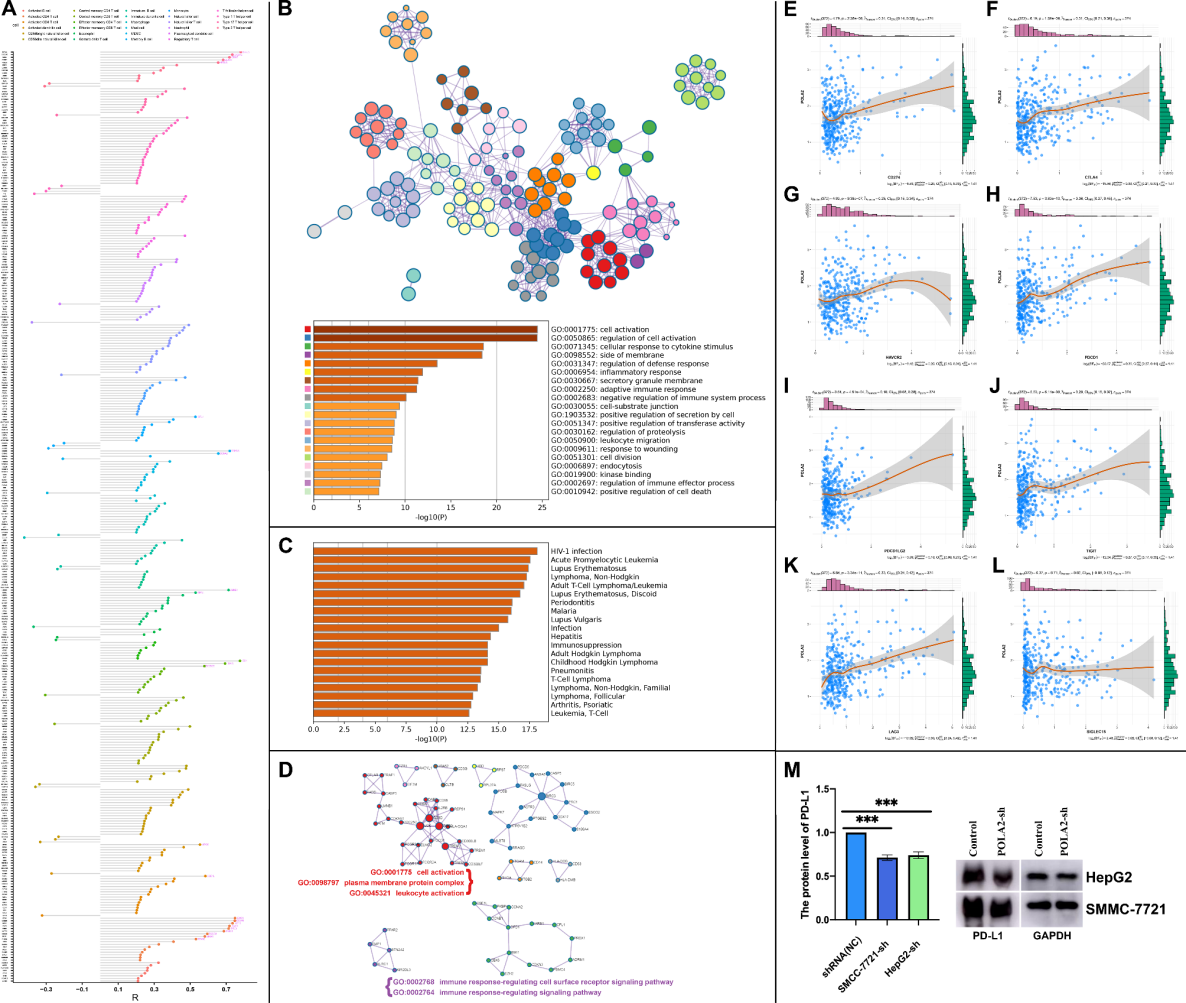
POLA2 significantly correlated with infiltrating immune cell markers and immune checkpoints in HCC. The significantly POLA2 co-expressed immune cell markers; (**B**-**D**) GO and KEGG functional enrichment of POLA2-associated immune cell markers by Metascape; (**E**-**L**) POLA2 immune checkpoint correlation analysis. (**M**) Western blot results for PD-L1 in HepG2 and SMMC-7721 cells and the relative protein expression level change of PD-L1

The correlation between POLA2 and immune cell marker genes results showed that POLA2 owned a co-expression relationship with 355 immune cell-related genes (|R|> 0.2, p < 0.01), and 304 genes showed a positive association. Among these co-expressed genes, the top significant related genes all showed positive relationship and list as follow (p < 0.01; r > 0.4): BIRC5, CDC25C, CDC7, CENPF, HELLS (Type 2 T helper cell); CFL1 (Monocyte); CDKN3, CCNA2 (Memory B cell); LMNB1, DBNL (Gamma delta T cell); EZH, TIPIN, EXOSC9 (Effector memory CD4 T cell); XPO6 (Activated dendritic cell); CSE1L (Activated CD8 T cell); EXO1, CCNB1, KIF11, NUF2, PRC1, KNTC1, ESCO2, BRIP1, RTKN2 (Activated CD4 T cell) (Fig. 9A).

Since POLA2 affected the infiltration of immune microenvironment in HCC, we studied the exact functional pathways of POLA2 by functional enrichment. The above mentioned 355 genes were enriched in cellular activity, plasma membrane protein complex and leukocyte activity, immune response-regulated cell surface receptor signaling pathway, and immune response-regulated signaling pathways by MEXPRESS analysis (Fig. 9B). Though the DisGeNET human diseases [33] results showed that POLA2 coexpressed genes also enriched to some immune related diseases, such as hepatitis, infection, different leukemia or Lymphoma, and so on (Fig. 9C).

The co-expression genes of POLA2 were related to the regulation of the immune effector process, and anticancer drugs that target immune checkpoints play an essential role in treating HCC [34]. Therefore, we further investigated the correlation between POLA2 and eight published immune checkpoints. The results shown that POLA2 significantly correlated with CD274, CTL-4, HAVCR2, PDCD1, PDCD1LG2, TIGIT, and LAG3 except SIGLEC15 (Fig. 9E-L). In current study, based on TCGA database analysis, we found a significant positive correlation between POLA2 and PDL-1 expression level in HCC. Then, knockdown of POLA2 in hepatocellular carcinoma cell lines SMMC-7721 and HepG2 revealed that PD-L1 protein level were also consistently suppressed. These results indicated that POLA2 might affect PD-L1 expression in HCC(Fig. 9M). And possibly due to the fact that POLA2, a member of the DNA polymerase family, promoted the production of PD-L1 in HCC by direct or indirect manners. In conclusion, the above studies demonstrated that POLA2 may play an important role in the immunity of HCC.

## Discussion

DNA polymerase α, one of the DNA polymerases, plays an important role in cell division and proliferation, tumorigenesis, and disease progression. Many studies have shown that abnormal expression of POLAs promoted tumor progression [35, 36]. We found that both POLA1 and POLA2 were highly expressed in HCC, and the high levels of POLA1 and POLA2 were associated with short OS in HCC patients. This was consistent with previous reports [37]. Furthermore, we found that both POLA1 and POLA2 were all significantly associated with T Stage and Grade of HCC. Besides, POLA2 was also significantly associated with the M stage of HCC. This correlation might suggest that POLA2 expression was associated with distant metastasis of HCC. Then we went through in vitro experiments and found similar conclusions.

Cancer cells migration or invasion play important role in initiating HCC metastasis and lead to less effective treatment outcomes [38, 39]. Our present data showed that POLA2 down regulation inhibited the growth, migration and invasion of HCC cells and further mitigated the progression of HCC. To gain a validation and deeper understanding of POLA2 prognostic role, we recruited 24 HCC patients. Both in mRNA and in protein levels, the expression of POLA2 were all significantly increased in HCC compared with paracancerous tissues. In addition, POLA2 expression also correlated with tumor size, differentiation, and Ki-67 expression in the tumors. Our results suggest that POLA2 may influence the proliferation of HCC cells, which was subsequently validated by vitro experiments. In animal experiments we found that after POLA2 knockdown, both tumor size, weight, and ki-67 expression level of tumors were suppressed. These findings suggested that POLA2 might be a novel marker for assessing the prognosis of patients with HCC.

Previous studies found that POLA2 was closely related to mutation of genes and repair of gene damage [3]. Our study found that HCC patients with high expression level of POLA2 showed significantly high mutation rate and TMB. Genes with significantly different mutations in the high and low POLA2 expression groups were: TP53, KIF19, CTNNB1, and RB1. TP53 is a relatively common mutation in HCC [40]. The mutation would affect the progression and prognosis of HCC [41]. Mutant TP53 induced cells that undergo DNA damage to escape apoptosis [42] and, in turn, evolved into cancer cells. In addition, it had been shown that mutated TP53 down-regulates the response of immune cells in the HCC microenvironment to cancer cells [43]. KIF19 was associated with the progression and prognosis of HCC [44]. Interestingly, CTNNB1 was the only one with a significant increased mutation rates in the POLA2 low-expression group, CTNNB1 mutation levels approximately ranged from 19 to 26% [45] in HCC. Mutations of CTNNB1 in liver cancer were often associated with the absence of immune cells in the tumor microenvironment [4]. A study shown that the tumor microenvironment lacked T cells in CTNNB1 mutated liver cancer [46]. RB1 dysfunction was often associated with HCC development and increased the efficacy of anticancer drugs. TTN was associated with HCC progression and can promote proliferation, migration and invasion of HCC cells [47]; MUC16 mutations were closely associated with HCC cell metabolism and immunity [48]; PCLO is associated with immune infiltration in HCC [49]. These studies indicated that mutations caused by high expression of POLA2 might also cause liver cancer progression.

Another important aspect of this study was the effect of POLA2 on the HCC microenvironment. Tumor infiltration by immune cells is linked with HCC prognosis and outcome [50, 51]. Therefore, infiltrating immune cells in tumors can be a therapeutic target for HCC treatments. Therefore, we analyzed the correlation between POLA2 and the infiltration of immune cells in HCC. The results revealed that POLA2 was positively correlated with T cell CD4 + Th1, T cell CD4 + Th2, common lymphoid progenitor and was negatively correlated with hematopoietic cells, stroma score, endothelial cell, and macrophage M2 in HCC. Studies had shown that dysregulation of T cell CD4 + Th1 and T cell CD4 + Th2 ratios in hepatocellular carcinogenesis and prognosis [52, 53]. Moreover, hematopoietic cells with unique NK repertories and functions to protect the liver cells from transformation to malignant tumors [54]. Enrichment analysis of immune genes associated with POLA2 expression in HCC revealed that POLA2 played an important role in the immune process.

Recently, immune checkpoint inhibitors (anti-PD-1, anti-PD-L1, and anti-CTLA-4 antibodies) had exhibited potential therapeutic effects for advanced HCC [55–57]. In addition, data from the phase I/II CheckMate-150 trial were presented at the 2022 annual meeting of the American Society of Clinical Oncology (ASCO 2022), demonstrating that the combination of atezolizumab (PD-L1 antibody) and bevacizumab (VEGF antibody) produced a median OS of 19.2 months vs. 13.4 months with sorafenib, representing a median 5.8-month increase [58]. These results indicated that immunotherapy had potential and surprising effects against HCC. Our study found that POLA2 was associated with immune checkpoints such as CD274, CTL-4, HAVCR2, PDCD1, PDCD1LG2, TIGIT, and LAG3. Among them, drugs targeted against PD-1 and PD-L1 have been widely used in treating HCC. Then, we verified that PD-L1 expressions were also reduced to different degrees after knocking down POLA2 in SMMC-7721 and HepG2 cell lines.The above results demonstrated a vital role of POLA2 in HCC tumor immunity, which was expected to be a prognostic marker for immunotherapy in HCC treatment.

## Conclusions

High expression of POLA2 was correlated with malignant phenotypes and worse outcomes in HCC. Mutation analysis revealed that POLA2 was associated with gene mutation in HCC. In addition, POLA2 was involved in regulating several immune pathways, and correlated with tumor immune microenvironment and the immune checkpoints expression. Consequently, our study suggested that POLA2 might be a promising prognostic and immunotherapeutic prediction marker for HCC.

## Electronic supplementary material

Below is the link to the electronic supplementary material.

Additional File 1: POLA2WB Raw Data.

Additional Figure S1: The high expression of POLA1 and POLA2 in hepatocellular carcinoma analyzed by HCCDB.

Additional Figure S2: (A) Co-expression analysis of POLA1 and POLA2; (B) ROC diagnostic curve of POLA1 and POLA2 on hepatocellular carcinoma; (C) Combination Kaplan-Meier analysis of the prognostic impact of POLA1 and POLA2 HCC based on overall survival time.

Additional Figure S3: The colony formation assay of HepG2, SMMC-7721 cells were transfected with shPOLA2 and control vectors.

Additional Figure S4: Immune cell type expression analysis results of POLA2 based on HPA, Monaco, and Schmiedel dateset.

## Data Availability

The data that support the findings of this study are openly available in the web site of University of California, Santa Cruz Xena [(https://xenabrowser.net/datapages/)] and TIMER 2.0 database (http://timer.cistrome.org/).

## List of abbreviations

HCC: Hepatocellular carcinoma
TCGA: The Cancer Genome Atlas
AFP: alpha-fetoprotein
FPKM: Fragments per kilobase of exon per million reads mapped
IHC: Immunohistochemical staining
RIPA: radio-immunoprecipitation assay
TMB: tumor mutation burden
