# Supplementary material for: Overexpression of POLA2 in hepatocellular carcinoma is involved in immune infiltration and predicts a poor prognosis
Source: Cancer Cell Int. 2023 Jul 14;23:138. doi: 10.1186/s12935-023-02949-z (PMC10349470; doi:10.1186/s12935-023-02949-z)

POLA2：66KD PATIENTS


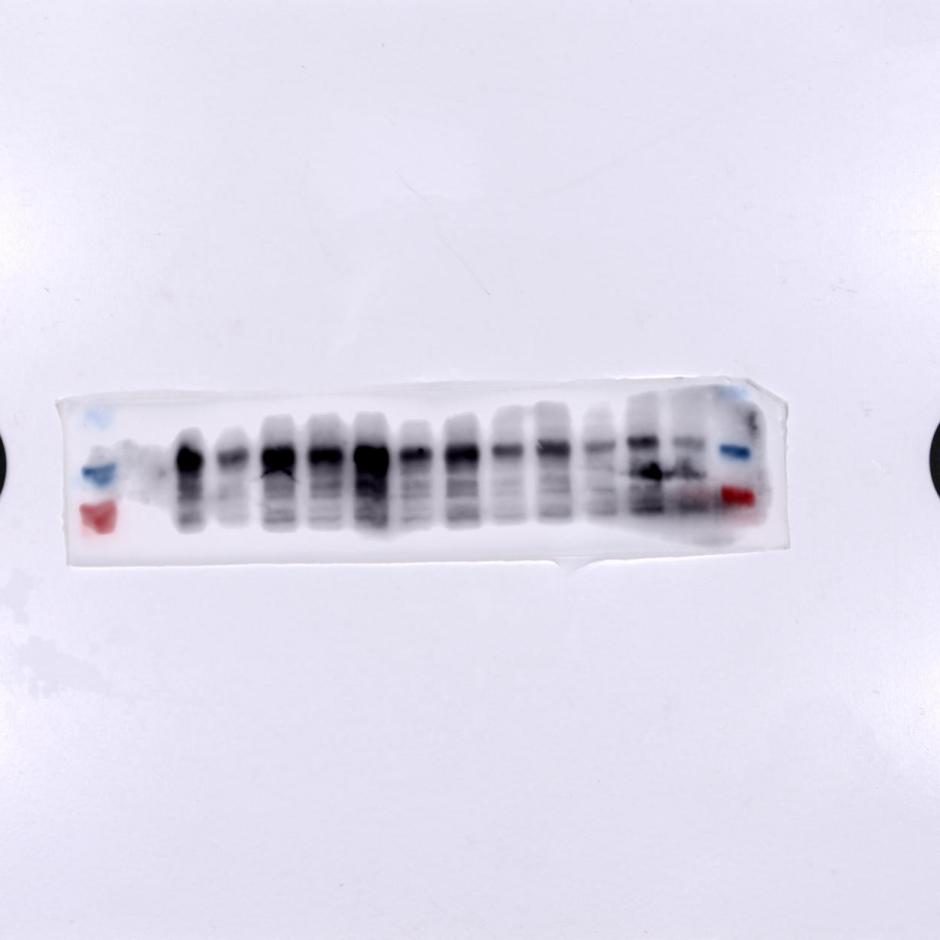


←55KD

←70KD

GAPDH：37KD PATIENTS


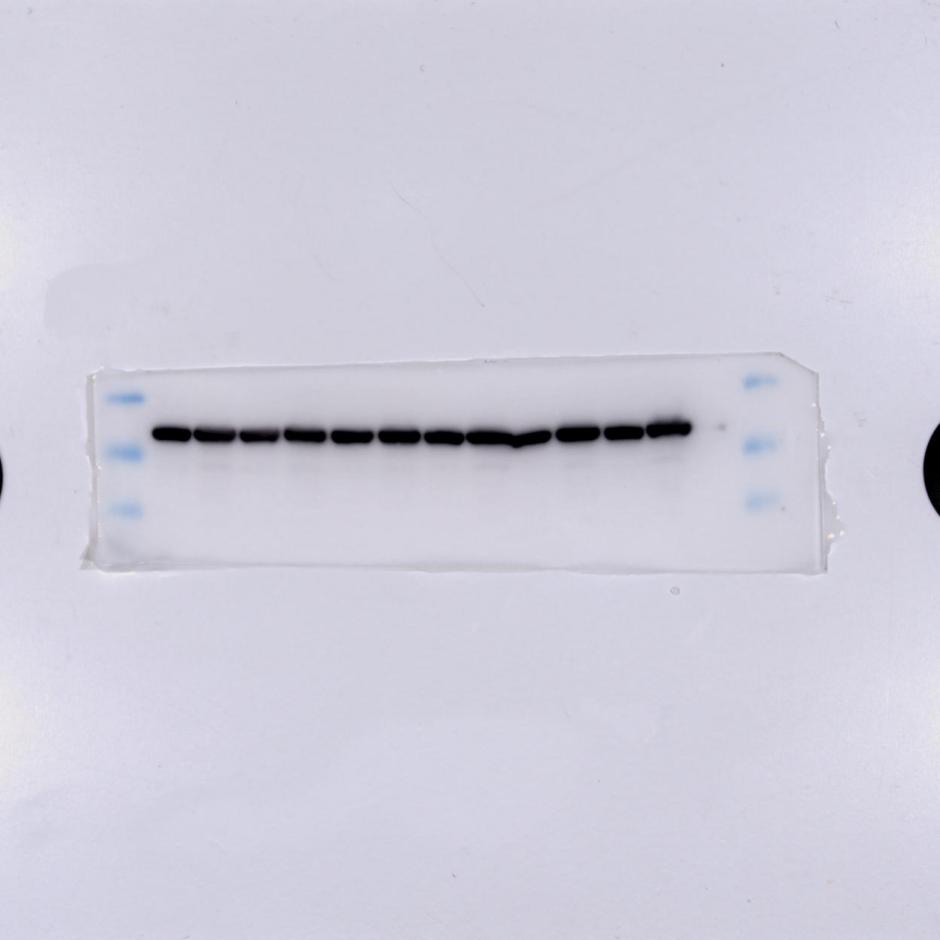


←40KD

←35KD

POLA2：40-65KD SMC-7721 AND HEPG2


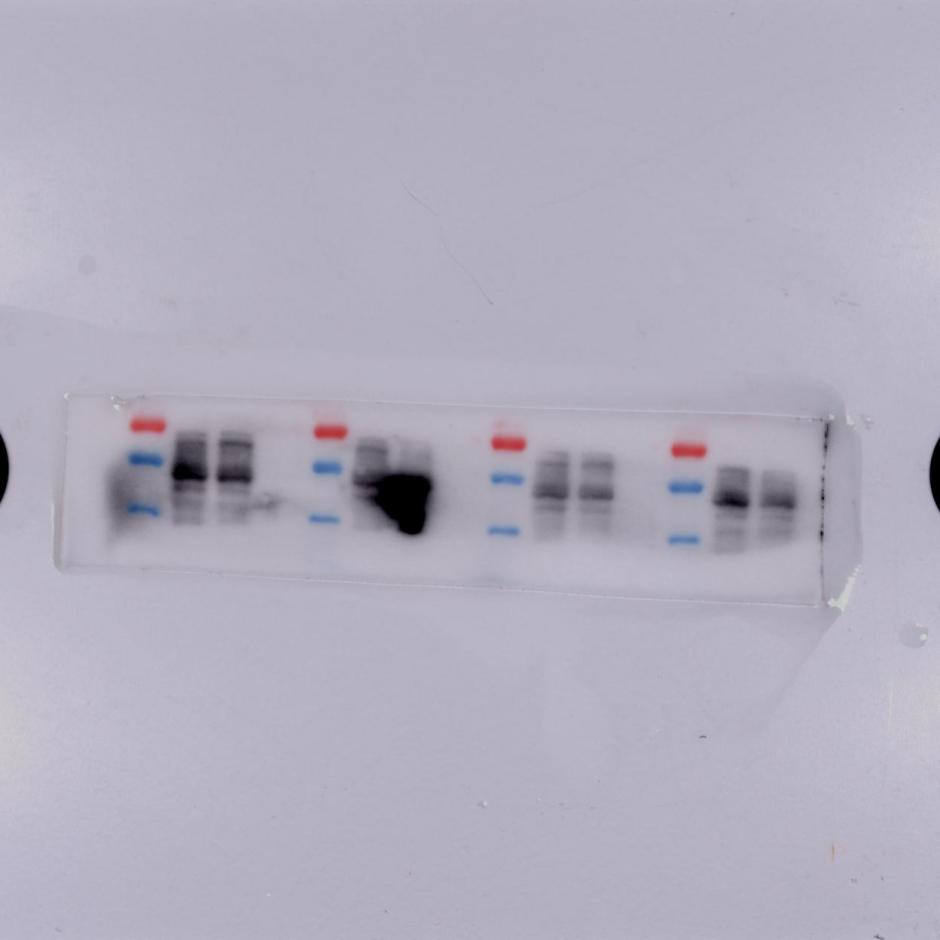


70KD→

55KD→

GAPDH：37KD SMC-7721 AND HEPG2


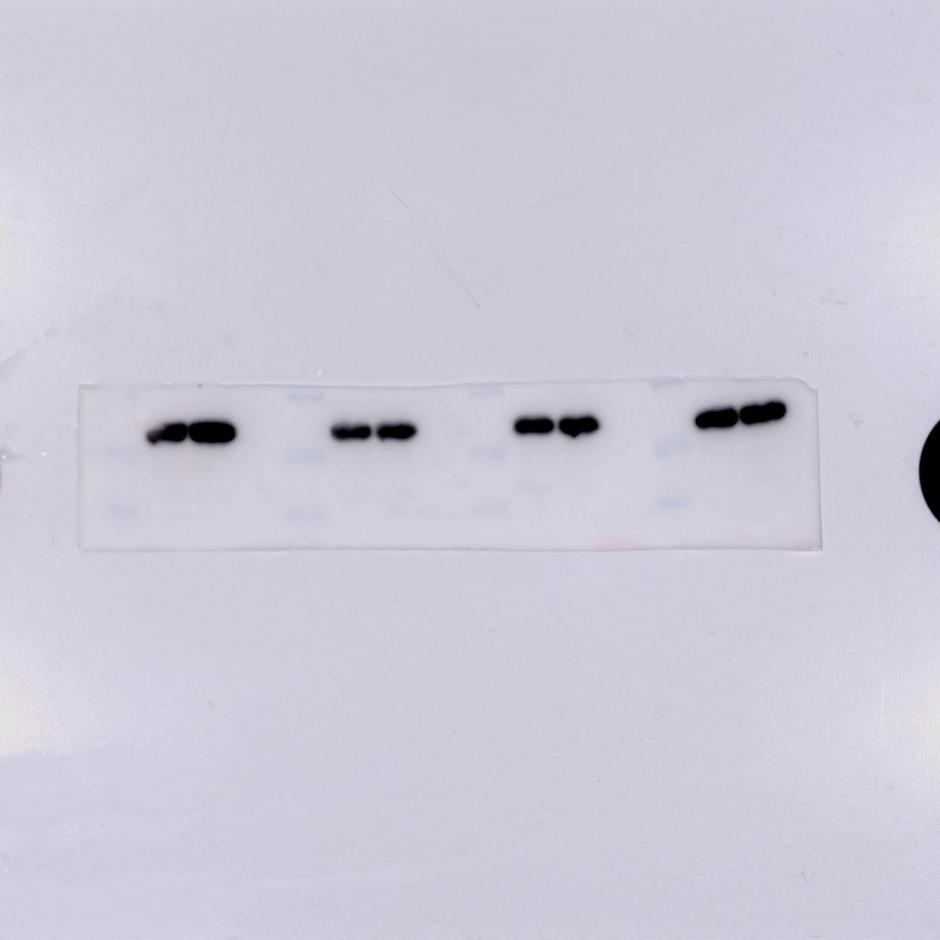


40KD→

35KD→

PD-L1：40-60KD HepG2


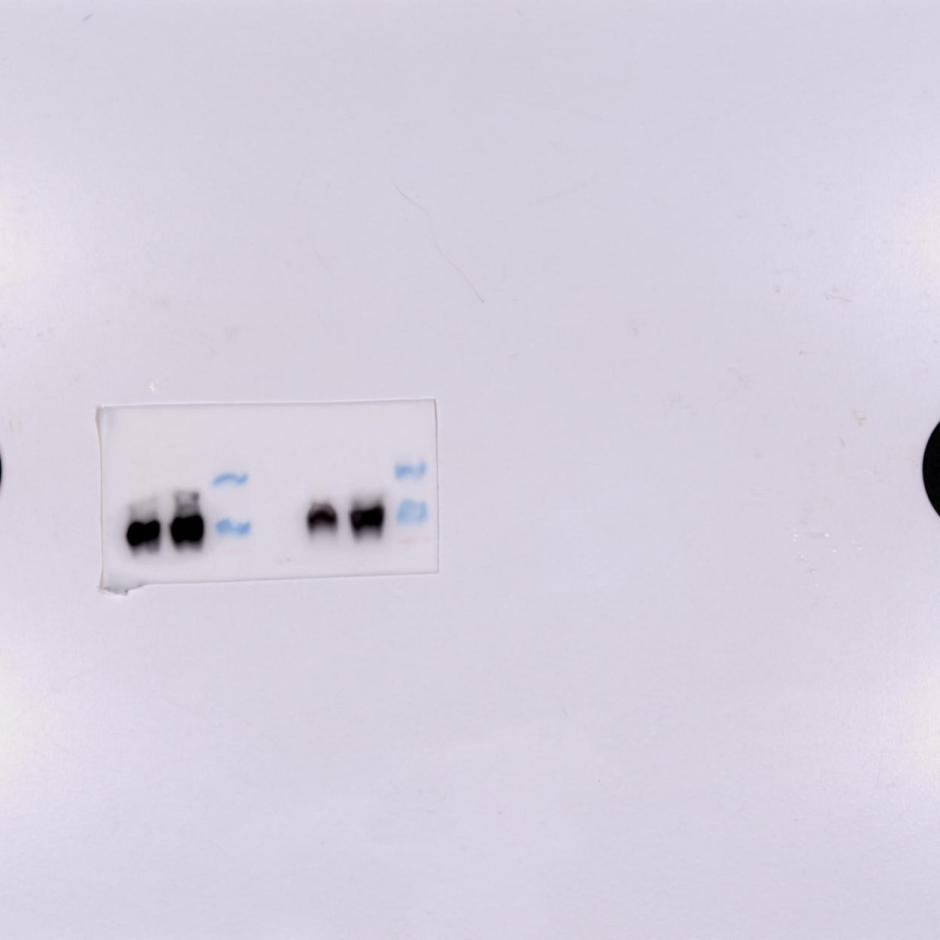


40KD→

55KD→

PD-L1：40-60KD SMC-7721


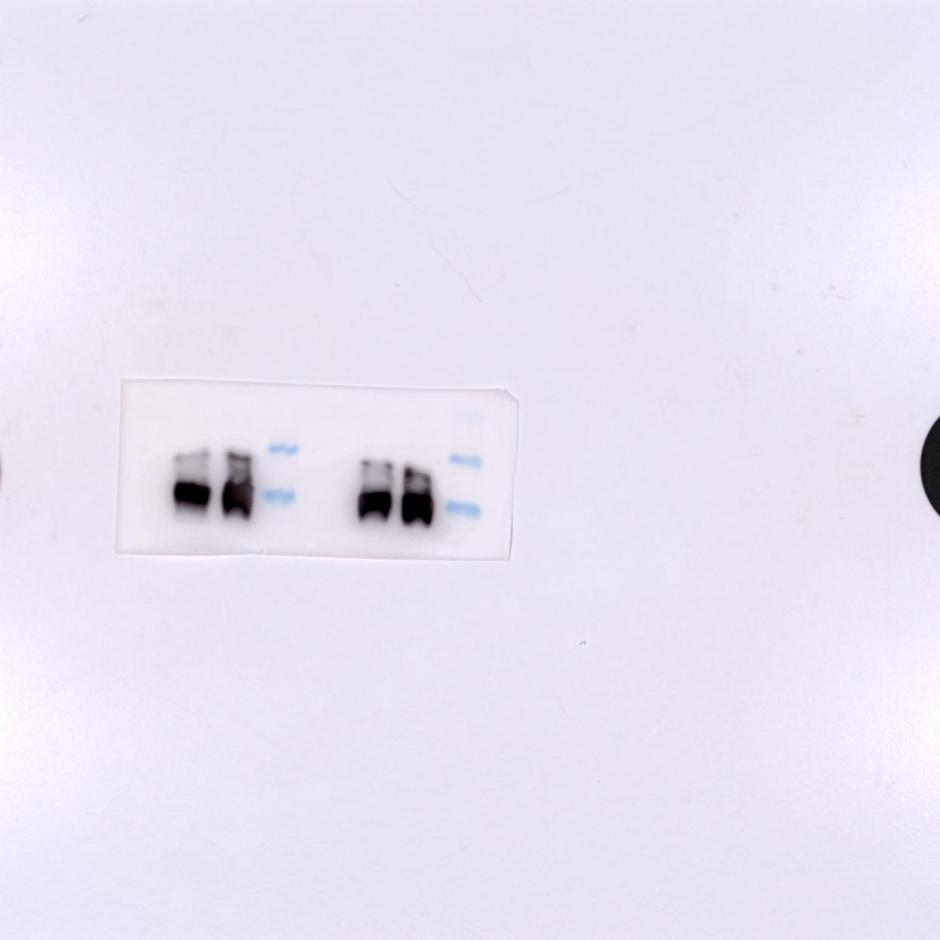


40KD→

55KD→

GAPDH：37KD SMMC-7721


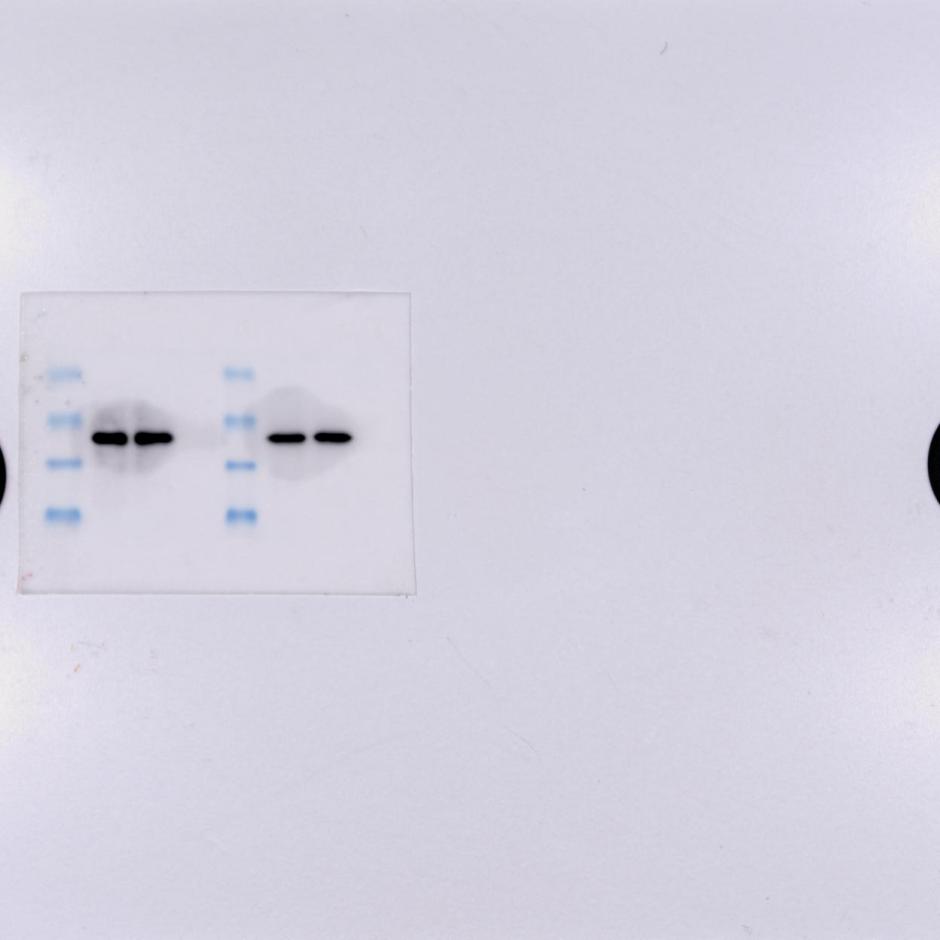


35KD→

40KD→

GAPDH：37KD HEPG2


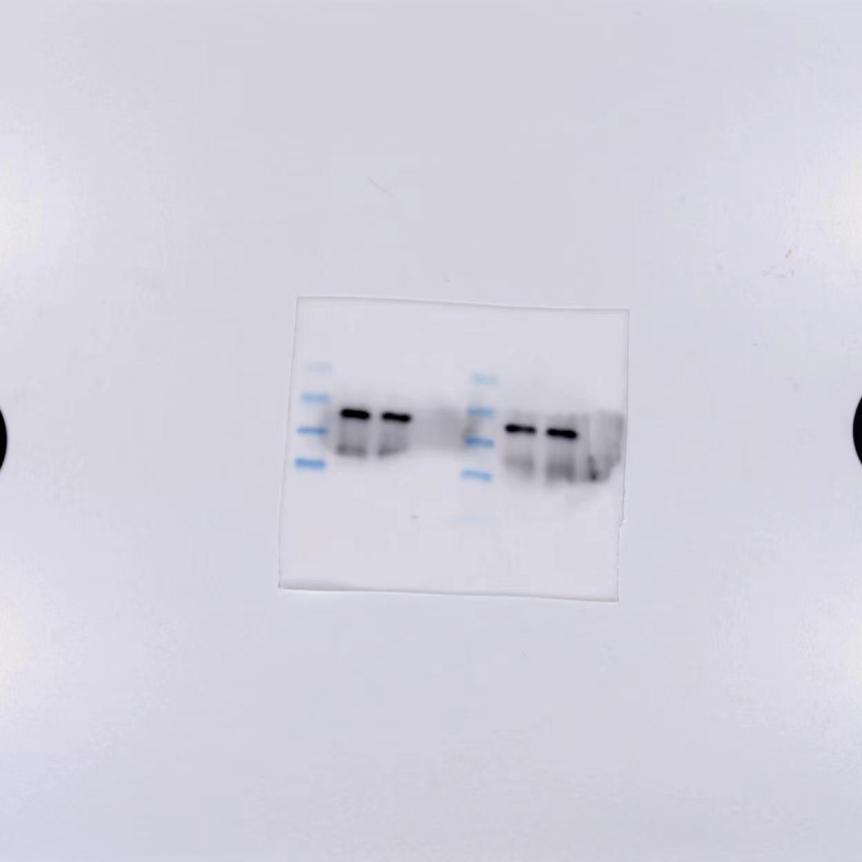


40kd

35kd

Color marker


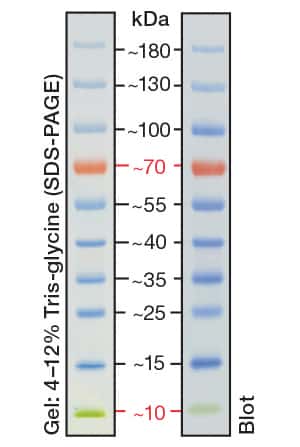

Supplement: Supplementary file 1 — Additional File 1: POLA2WB Raw Data. [file 12935_2023_2949_MOESM1_ESM.docx]
