# Supplementary figures and images for: Overexpression of POLA2 in hepatocellular carcinoma is involved in immune infiltration and predicts a poor prognosis
Source: Cancer Cell Int. 2023 Jul 14;23:138. doi: 10.1186/s12935-023-02949-z (PMC10349470; doi:10.1186/s12935-023-02949-z)

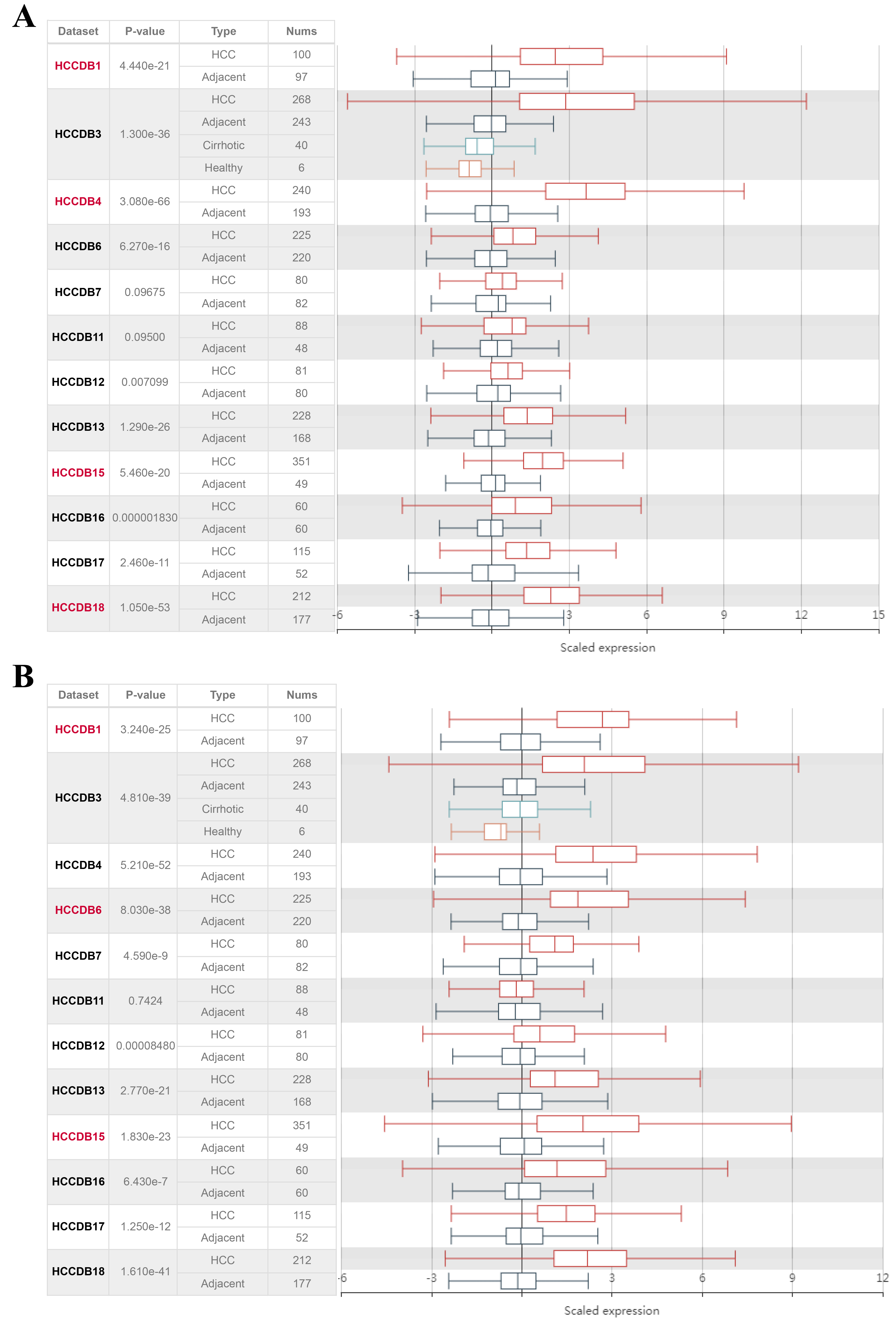

Supplement: Supplementary file 2 — Additional Figure S1: The high expression of POLA1 and POLA2 in hepatocellular carcinoma analyzed by HCCDB. [file 12935_2023_2949_MOESM2_ESM.png]

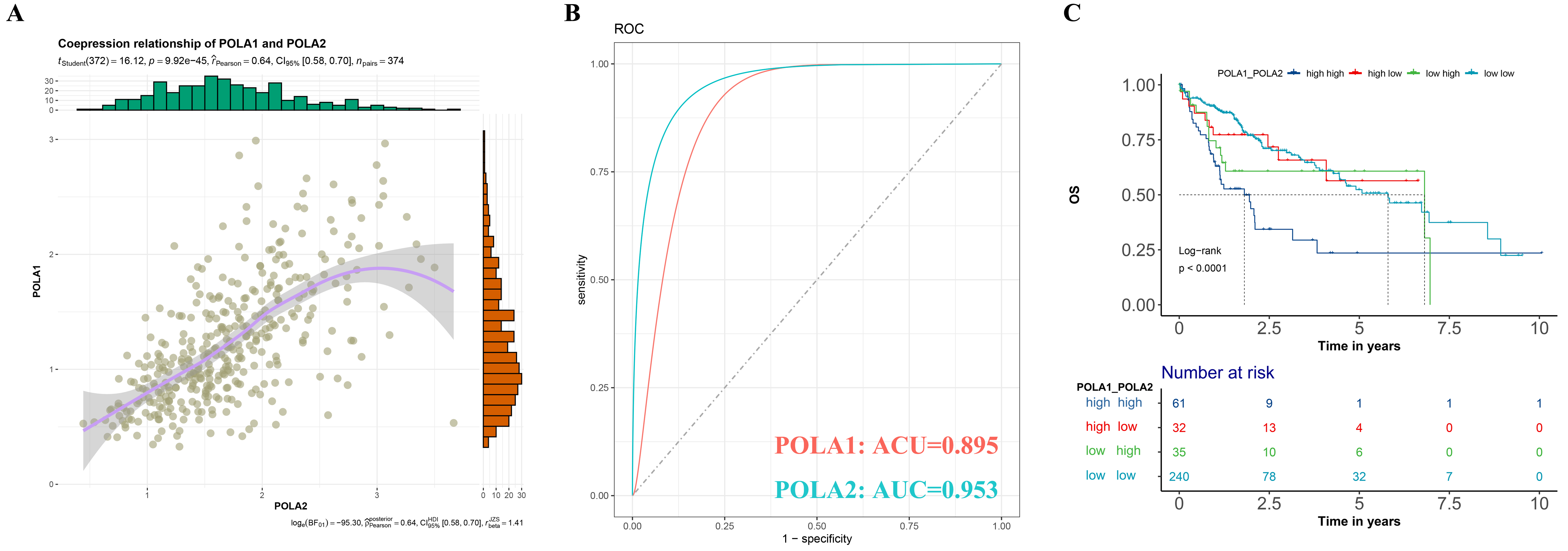

Supplement: Supplementary file 3 — Additional Figure S2: (A) Co-expression analysis of POLA1 and POLA2; (B) ROC diagnostic curve of POLA1 and POLA2 on hepatocellular carcinoma; (C) Combination Kaplan-Meier analysis of the prognostic impact of POLA1 and POLA2 HCC based on overall survival time. [file 12935_2023_2949_MOESM3_ESM.png]

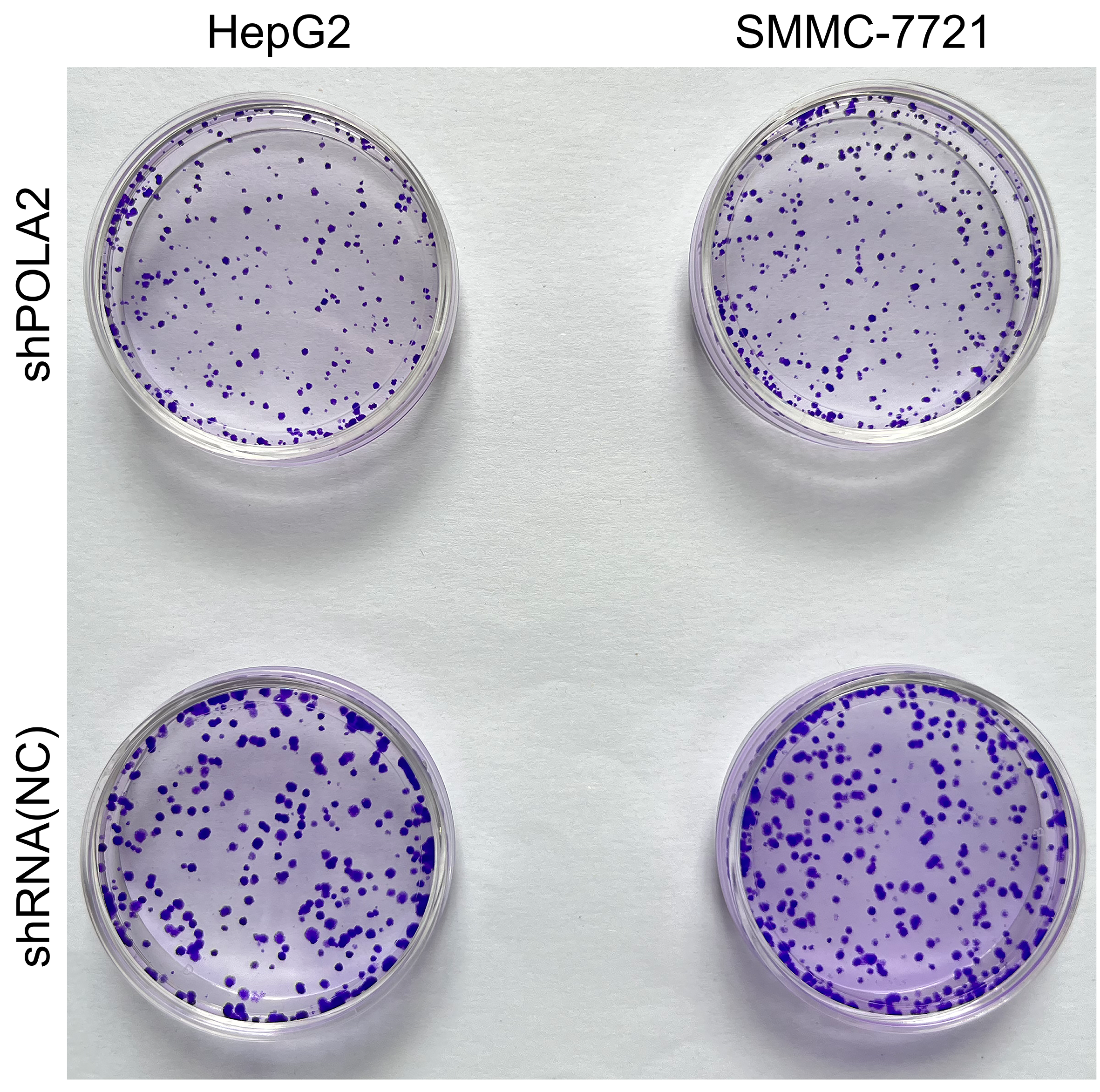

Supplement: Supplementary file 4 — Additional Figure S3: The colony formation assay of HepG2, SMMC-7721 cells were transfected with shPOLA2 and control vectors. [file 12935_2023_2949_MOESM4_ESM.png]
